# Different Decellularization Methods in Bovine Lung Tissue Reveals Distinct Biochemical Composition, Stiffness, and Viscoelasticity in Reconstituted Hydrogels

**DOI:** 10.1021/acsabm.2c00968

**Published:** 2023-02-02

**Authors:** Alican Kuşoğlu, Kardelen Yangın, Sena N. Özkan, Sevgi Sarıca, Deniz Örnek, Nuriye Solcan, İsmail C. Karaoğlu, Seda Kızılel, Pınar Bulutay, Pınar Fırat, Suat Erus, Serhan Tanju, Şükrü Dilege, Ece Öztürk

**Affiliations:** † Engineered Cancer and Organ Models Laboratory, Koç University, Istanbul 34450, Turkey; ‡ Research Center for Translational Medicine (KUTTAM), Koç University, Istanbul 34450, Turkey; § Department of Medical Biology, School of Medicine, Koç University, Istanbul 34450, Turkey; ∥ Department of Thoracic Surgery, School of Medicine, Koç University, Istanbul 34450, Turkey; ⊥ Department of Pathology, School of Medicine, Koç University, Istanbul 34450, Turkey; # Chemical and Biological Engineering, Koç University, Istanbul 34450, Turkey

**Keywords:** decellularization, lung hydrogels, tissue engineering, lung cancer, extracellular matrix

## Abstract

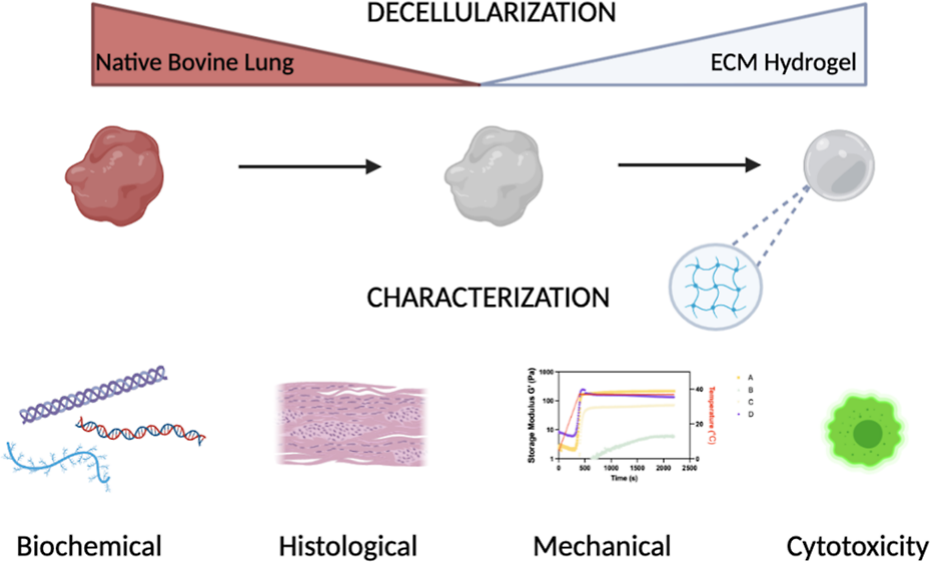

Extracellular matrix (ECM)-derived hydrogels are in demand for use in lung tissue engineering to mimic the native microenvironment of cells in vitro. Decellularization of native tissues has been pursued for preserving organotypic ECM while eliminating cellular content and reconstitution into scaffolds which allows re-cellularization for modeling homeostasis, regeneration, or diseases. Achieving mechanical stability and understanding the effects of the decellularization process on mechanical parameters of the reconstituted ECM hydrogels present a challenge in the field. Stiffness and viscoelasticity are important characteristics of tissue mechanics that regulate crucial cellular processes and their in vitro representation in engineered models is a current aspiration. The effect of decellularization on viscoelastic properties of resulting ECM hydrogels has not yet been addressed. The aim of this study was to establish bovine lung tissue decellularization for the first time via pursuing four different protocols and characterization of reconstituted decellularized lung ECM hydrogels for biochemical and mechanical properties. Our data reveal that bovine lungs provide a reproducible alternative to human lungs for disease modeling with optimal retention of ECM components upon decellularization. We demonstrate that the decellularization method significantly affects ECM content, stiffness, and viscoelastic properties of resulting hydrogels. Lastly, we examined the impact of these aspects on viability, morphology, and growth of lung cancer cells, healthy bronchial epithelial cells, and patient-derived lung organoids.

## 1 Introduction

The extracellular matrix (ECM) is a supramolecular entity composed of different building blocks organized specifically for every tissue. Cellular processes are dramatically affected by matrix properties because there is constant interaction between cells and their microenvironment. Likewise, the ECM undergoes both mechanical and biochemical changes mediated by the cells embedded within. This bi-directional cell-matrix interaction is known as dynamic reciprocity and holds great importance in regulating physiological and pathological processes in a tissue-specific manner.^1^ Biochemical composition and mechanical properties of the tissue ECM have been emphasized for their important regulatory role on cellular behavior for a long time.^2−4^ Numerous pioneering studies have demonstrated how tissue stiffness governs a plethora of processes including differentiation, proliferation, apoptosis, malignancy, and drug resistance.^5−9^ Mechanosensitive receptors such as integrins act as bridges between the ECM and cytoskeleton whose bi-directional stimulation controls homeostasis of tissues and disruption of their interaction takes role in pathological conditions.^10^ While tissue stiffness has been established in the field as a vital parameter regarding the mechanical microenvironment, studies have relied on the assumption of pure elasticity of tissue matrices. However, native tissue ECMs are viscoelastic and demonstrate time-dependent deformation upon stress application.^11^ Recently, ECM viscoelasticity has gained pronounced appreciation for its role in mechanotransduction and engineered tissue models with tunable viscoelasticity have revealed its contribution to homeostasis, growth, differentiation, and malignant progression.^12^

Given the importance of ECM in controlling cellular behavior, creating models where native ECM characteristics are faithfully recapitulated has been aspired by the tissue engineering field. Decellularization of tissues and organs has gained attention with the ability of preserving native tissue matrices while eliminating cellular content and reconstitution into forms such as hydrogels which allows re-cellularization or cell embedding.^13,14^ Several physical methods such as freeze-thawing,^15^ vigorous agitation,^13^ application of high hydrostatic pressures^16^ or supercritical CO_2_;^17^ and chemical methods utilizing sodium dodecyl sulfate (SDS),^18^ sodium deoxycholate (SDC),^19^ Triton-X-100,^14^ sodium hydroxide,^20^ 3-[(3-cholamidopropyl) dimethylammonium]-1-propanesulfonate (CHAPS),^21,22^ peracetic acid,^23,24^ and ammonium hydroxide^25^ are used in combination with trypsin,^26^ dispase,^27^ or DNAse^28^ treatments in decellularization approaches. These methods demonstrated comparative strengths and weaknesses, but loss of key biochemical content [such as sulfated glycosaminoglycans (sGAGs)], mechanical instability, and batch-to-batch variability have been the dominant disadvantages in the field.^29,30^ A thorough characterization of the effect of decellularization methods on biochemical and mechanical aspects of reconstituted scaffolds including viscoelasticity and stiffness is key to improve engineered tissue models derived from native matrices.

Lung tissue engineering aims to build in vitro human models that can successfully biomimic the native lung microenvironment using synthetic or natural materials in order to address pulmonary pathologies with low clinical output such as cancer and chronic obstructive pulmonary disease.^31−33^ The use of decellularized matrices is highly promising with respect to the preservation of organ-specific ECM composition and presentation of cell instructive cues in disease modeling.^34^ The influence of matrix components on lung epithelium have been shown within the contexts of differentiation and structural organization.^35,36^ Several studies have demonstrated successful decellularization of lung tissue derived from species such as rat,^20^ porcine,^34,37,38^ and human.^34^ Decellularization of porcine lung with SDS, CHAPS, and three-step methods were compared for ECM content and support of cellular growth.^37^ Effects of parameters such as digestion time on biochemical and gelation properties of lung dECM hydrogels and subsequent cellular response have been identified.^19^ Tissue-specific composition of biomolecules such as collagen, elastin, sGAGs, laminin, and fibronectin is the main contributor of the mechanical properties of the ECM which is altered in disease conditions. Healthy lung tissue is viscoelastic and has a Young’s modulus between 1 and 5 kPa that aberrantly increases in pathological conditions such as fibrosis.^39^ Even though changes in stiffness of certain tissues have been characterized thoroughly, effects of altered viscoelasticity on disease conditions are not yet understood clearly.^40^ Understanding the effect of viscoelasticity on cellular behavior is a rather new pursuit in tissue engineering. Extensive characterization of the compositional differences due to decellularization techniques and how they reflect on distinct mechanical properties of lung dECM remains a gap in the field. This calls for overarching studies where different decellularization methods are characterized for optimal recapitulation of organotypic features.

In this study, we established decellularization of lung tissue from bovine pursuing several protocols and performed comprehensive analyses on resulting biochemical and mechanical properties of dECM. We fabricated reconstituted hydrogels from lung dECM via ability of thermal crosslinking, characterized gelation kinetics, hydrogel stiffness, and viscoelastic properties. Our data reveal method-based differences in the biochemical composition of dECM material and show that hydrogel stiffness and viscoelasticity change dramatically depending on the decellularization process. Lung adenocarcinoma, normal bronchial epithelial cells, and patient-derived healthy lung organoids were encapsulated in hydrogels and monitored for differences in growth and morphology due to varying methods. Overall, our study provides a translation of decellularization methods to dECM hydrogels’ composition, stiffness, viscoelasticity, and subsequent cellular responses.

## 2 Experimental Section

### 2.1 Harvest of Organs and Tissue Preparation

Bovine lung tissues were procured from a local slaughterhouse. Native human lung samples were collected with Koc University Institutional Review Board (2020.001.IRB2.001) ethics approval and consent of participants undergoing lobectomy as part of their clinical care. Tissue samples were dissected into smaller pieces, minced, and extensively washed with ultrapure water supplemented with 2% penicillin/streptomycin (P/S). Some portions of the native lung samples were stored as wet tissues at −20 °C for biochemical characterization assays.

### 2.2 Decellularization of Lung Tissues

Bovine lungs were decellularized using four different protocols.

#### 2.2.1 Freeze Thaw

Lung tissue pieces were immersed in 2% iodine solution (Sigma, 03002) in sterile dH_2_O for 1 min followed by two-step wash in sterile dH_2_O. Then, tissue pieces immersed in sterile dH_2_O in falcon tubes were subjected to five manual freeze-thaw cycles of 2 min freezing in liquid nitrogen and 10 min thawing in a 37 °C water bath. Next, tissues were treated with 10 U/mL DNase (Sigma, DN25) in 10 mM MgCl_2_ buffer (pH 7.5) for 1 h at 37 °C under constant shake. Finally, tissue pieces were thoroughly washed with sterile dH_2_O for 72 h under gentle rotation, with solution exchange every 24 h.

#### 2.2.2 Peracetic Acid

Minced tissue samples were exposed to 3% peracetic acid (Sigma, 433241) incubation at 37 °C under constant shake for 3 h. Then, the acid is washed off with phosphate-buffered saline (PBS) several times and tissue pieces were immersed in 1% Triton-X-100 (Merck, 112298) solution for 24 h under constant rotation at 4 °C. Detergent was rinsed off extensively and 2% SDC (Sigma, D6750) incubation was implemented for 24 h at 4 °C. Next, tissues were treated with 10 U/mL DNase in 10 mM MgCl_2_ buffer (pH 7.5) for 1 h at 37 °C under constant shake.

#### 2.2.3 Sodium Dodecyl Sulfate

Minced tissues were washed with sterile dH_2_O for 45 min with stirring and the tissues were strained in an autoclaved fine mesh strainer. Detergent treatment was implemented to incubating tissues in 1% SDS solution (Sigma, L3771) for 26 h with a solution replenishment after 2 h. In order to rinse off all the residual detergent, tissues were extensively washed with sterile dH_2_O. Next, tissues were treated with 10 U/mL DNase in 10 mM MgCl_2_ buffer (pH 7.5) for 1 h at 37 °C under constant shake.

#### 2.2.4 TritonX

Minced tissue pieces were immersed in 1% Triton-X-100 solution for 72 h under constant rotation at 4 °C and solutions were replenished every 24 h. Next, tissues were treated with 10 U/mL DNase in 10 mM MgCl_2_ buffer (pH 7.5) for 1 h at 37 °C under constant shake. Finally, tissue pieces were thoroughly washed with sterile dH_2_O for 72 h under gentle rotation, with solution exchange every 24 h.

Following the decellularization process with designated protocols, some portions of the samples were stored as wet tissues in −20 °C for biochemical characterization assays. The rest of the dECM samples were stored at −80 °C for a day and then lyophilized. Lyophilized samples were cryo-milled into a fine powder form.

### 2.3 Pepsin Digestion

dECM samples in powder form were digested at designated concentrations (10, 15, and 20 mg/mL, w/v) in pepsin (Sigma, P6887) solution (1 mg/mL pepsin in 0.01 M HCl) (Merck, 100317) at room temperature under constant stirring for 48 h. Upon completion, a part of all samples were spared as total digests and the remaining samples were centrifuged at 5000*g* for 10 min. Supernatants were collected, labeled, and used as soluble digests. Then, both total and soluble digests were neutralized and buffered to physiological conditions (pH 7, 1X PBS) by adding NaOH (Sigma, S5881) and 10X PBS. These pre-gel forms were stored at −20 °C for further studies.

### 2.4 dsDNA Quantification

dsDNA quantification was performed by Quant-iT PicoGreen dsDNA Assay Kit (Invitrogen, P7589) according to manufacturer’s specifications. DNA content of native human, native lung, and decellularized lung tissues were assessed. Briefly, 3 mg of dry tissue samples were digested in 125 μg/mL of papain (Sigma-P4762) buffer [400 mg of sodium acetate, 200 mg of ethylenediaminetetraacetic acid (EDTA), and 40 mg of cysteine in 50 mL of 0.2 M sodium phosphate buffer, pH 6.4] at 60 °C overnight. After digestion, samples were diluted in TE buffer (supplied by the kit) and mixed with Quant-iT PicoGreen reagent. Fluorescence was measured at 520 nm with excitation at 485 nm in a microplate reader. dsDNA content was quantified using a standard curve and normalized to sample weight.

### 2.5 Collagen Quantification

S2000 Sircol Insoluble Collagen Assay (Biocolor, UK) was used for quantification of collagen content in native and decellularized tissues. The Sircol dye in the kit content recognizes the tripeptide sequence [(gly-X-Y)*n*] of the collagen fibers and allows colorimetric measurement. The assay was performed by following the manufacturer’s instructions. Briefly, wet tissues (20–30 mg) were weighted, and native insoluble collagen was converted into denatured collagen through a mild acid and temperature treatment. Then, Sircol dye reagent was added onto test samples. Binding of the dye to tripeptide sequences formed a red precipitate. Unbound dye was washed away, and collagen bound dye was recovered with the alkali reagent. Colorimetric quantification of the denatured collagen was done via measuring the absorbance at 550 nm in a microplate reader and collagen content was normalized to wet weight of tissue samples.

### 2.6 Elastin Quantification

Elastin content in native and decellularized tissues were quantified by the Fastin Elastin Assay Kit (Biocolor, UK) according to manufacturer’s instructions. Wet tissues (15–20 mg) were weighed, and the elastin extraction was achieved via three consecutive hot oxalic acid (0.25 M) incubations. These three cycles of incubation are required to ensure complete elastin extraction from the lung tissues. In each cycle of the extraction, supernatants were collected upon centrifugation at 10,000*g* and then pooled. At least three samples were analyzed for each native and decellularized tissue sample. Elastin content was normalized to wet weight of the tissues.

### 2.7 sGAG Quantification

sGAG content in native and decellularized lung tissues were assessed by Blyscan sGAG Assay kit (Biocolor, UK) following manufacturer’s instructions. Briefly, 15–20 mg of wet tissue samples was digested in a solution containing 125 μg/mL of papain buffer (400 mg of sodium acetate, 200 mg of EDTA, and 40 mg of cysteine in 50 mL of 0.2 M sodium phosphate buffer, pH 6.4) at 65 °C overnight. Then, samples were mixed with the dye reagent, followed by dye retrieval and absorbance measurement at 656 nm in a microplate reader. sGAG content was normalized to wet weight of the tissues.

### 2.8 Histology and Immunofluorescence

Native and decellularized lung tissue samples were fixed with 3.7% formaldehyde solution (EMS) at 4 °C overnight. Fixed tissues were immersed in 30% sucrose overnight and then embedded in OCT (Tissue-Tek) and snap-frozen. 10 μm sections were obtained with a cryostat and mounted on glass slides. For DNA staining with Hoechst, slides were hydrated with PBS and stained for 15 min in 1 μg/mL Hoechst solution (Invitrogen) and visualized by fluorescence microscopy. Haematoxylin and eosin staining was performed to validate the absence of nuclei upon decellularization. Briefly, slides were hydrated and stained with Mayer’s haematoxylin (Merck) for 3 min, followed by a 3 min wash with tap water. Then, slides were immersed in 95% ethanol and stained with eosin solution (bright-slide) for 45 s. For collagen staining, Sirius Red (PolySciences) in a saturated aqueous solution of picric acid was used. Slides were immersed in Sirius red solution for 1 h and then rinsed in 0.5% acetic acid solution. Alcian blue staining was performed to visualize sGAGs. Slides were hydrated and stained with 1% Alcian blue (Sigma) in 3% acetic acid solution pH 2.5 for 30 min, followed by a 2 min wash with tap water. After staining, all slides were dehydrated with graded alcohol, mounted, and visualized by light microscopy.

### 2.9 Mechanical Characterization

Oscillatory rheology was performed using a Discovery HR-2 rheometer (TA instruments). 250 μL of neutralized dECM precursor solution was poured onto the lower plate which was pre-cooled to 4 °C and the 20 mm parallel plate was immediately lowered until the dECM solution fills the gap. Then, the lower plate was heated to 37 °C, and storage and loss moduli were measured over time with a fixed frequency of 0.5 Hz and 0.1% strain which were found to be in the linear viscoelastic range. When storage moduli of the sample reached an equilibrium state, a creep-recovery test was performed where 1 Pa shear stress was applied for 15 min and strain was measured; then, the sample was unloaded, and strain was recorded over time. All measurements were done in triplicates.

### 2.10 Cell Culture and Hydrogel Encapsulation

The human lung adenocarcinoma and bronchial epithelial cell lines, A549 (CCL-185) and BEAS-2B (CRL-9609), respectively, were purchased from American Type Culture Collection (ATCC) and cultured as monolayers in a growth medium comprising DMEM/F12 (Lonza) supplemented with 10% FBS and 1% P/S. For hydrogel embedding in dECM gels, cells were grown until 80% confluence, trypsinized, counted, and resuspended in designated dECM precursor solutions (kept at 4 °C) at a concentration of 1 × 10^5^ cells/mL. 30 μL cold cell-dECM mix was casted onto a single well on a 24-well plate and placed in the incubator (37 °C, 5% CO_2_) for 45 min for allowing gelation. Upon completion of gelation, the growth medium was carefully added and hydrogels were cultured up to 14 days in the incubator (37 °C, 5% CO_2_), while the medium was changed three times a week.

### 2.11 Patient-Derived Lung Organoids

Small pieces of non-neoplastic lung parenchyma derived from patients undergoing lobectomy were transported to the laboratory in a growth medium with 2% P/S. Samples were then cut into small pieces and washed three times until excess blood was washed away. Dissected tissues were incubated in 1 mg/mL collagenase solution (Sigma, C5138) in growth medium at 37 °C for 1 h with intermittent agitation. After incubation, the suspensions were repeatedly triturated by pipetting and passed through 70 μm cell strainers (BD Falcon). Then, cells were centrifuged at 300*g* for 5 min 4 °C, and the pellet was resuspended in Matrigel (Corning) and allowed to solidify on 12-well tissue culture plates for 30 min in the incubator (37 °C, 5% CO_2_). After gelation, pre-warmed PneumaCult-Ex Plus medium (STEMCELL) was added to each well. For passaging and dECM encapsulation, Matrigel hydrogels containing lung organoids were harvested using cold growth medium and then centrifuged at 300*g* for 5 min at 4 °C. Organoid pellets were then resuspended in 1 mL of TrypLE Express (Invitrogen) and incubated for 10 min at 37 °C for dissociation of organoids. After incubation, the growth medium was added and organoid suspension was centrifuged at 300*g* for 5 min. Pellets were then resuspended as equal concentrations in A-dECM and D-dECM as previously described, solutions were allowed to solidify for 45 min in the incubator (37 °C, 5% CO_2_). Lung organoids in dECM hydrogels were cultured for 10 days to monitor cell viability and growth.

### 2.12 In Vitro Cytocompatibility Assays

#### 2.12.1 Live/Dead Imaging

A549, BEAS-2B cells, and patient-derived lung organoids encapsulated in dECM gels were stained on designated time-points with calcein-AM (Invitrogen) and propidium iodide (PI) (Sigma) for viability assessment. Hydrogels were incubated in growth medium containing 2 μM calcein-AM and 30 μg/mL PI for 1 h at 37 °C, 5% CO_2_. After incubation, gels were rinsed three times with PBS and imaged immediately with fluorescence microscopy.

#### 2.12.2 CTG 3D Assay

A549 cells were encapsulated in dECM gels and cultured for 14 days in a growth medium. On days 1, 4, 7, 11, and 14 after plating, CellTiter-Glo 3D (Promega) reagent was added to cultures for 1 h and then culture media were collected and transferred into a 96-well plate and luminescence was measured in a microplate reader. All measurements were performed in triplicates. A growth curve was generated for each tested condition.

### 2.13 Statistics

All data were presented as means with standard deviation (sd). Statistics were performed with one-way ANOVA with Tukey tests for pairwise comparisons on multiple groups using GraphPad Prism 9, where a *p*-value of <0.05 was considered significant.

## 3 Results

### 3.1 Decellularization of Native Bovine Lung Tissue

**Figure 1:**
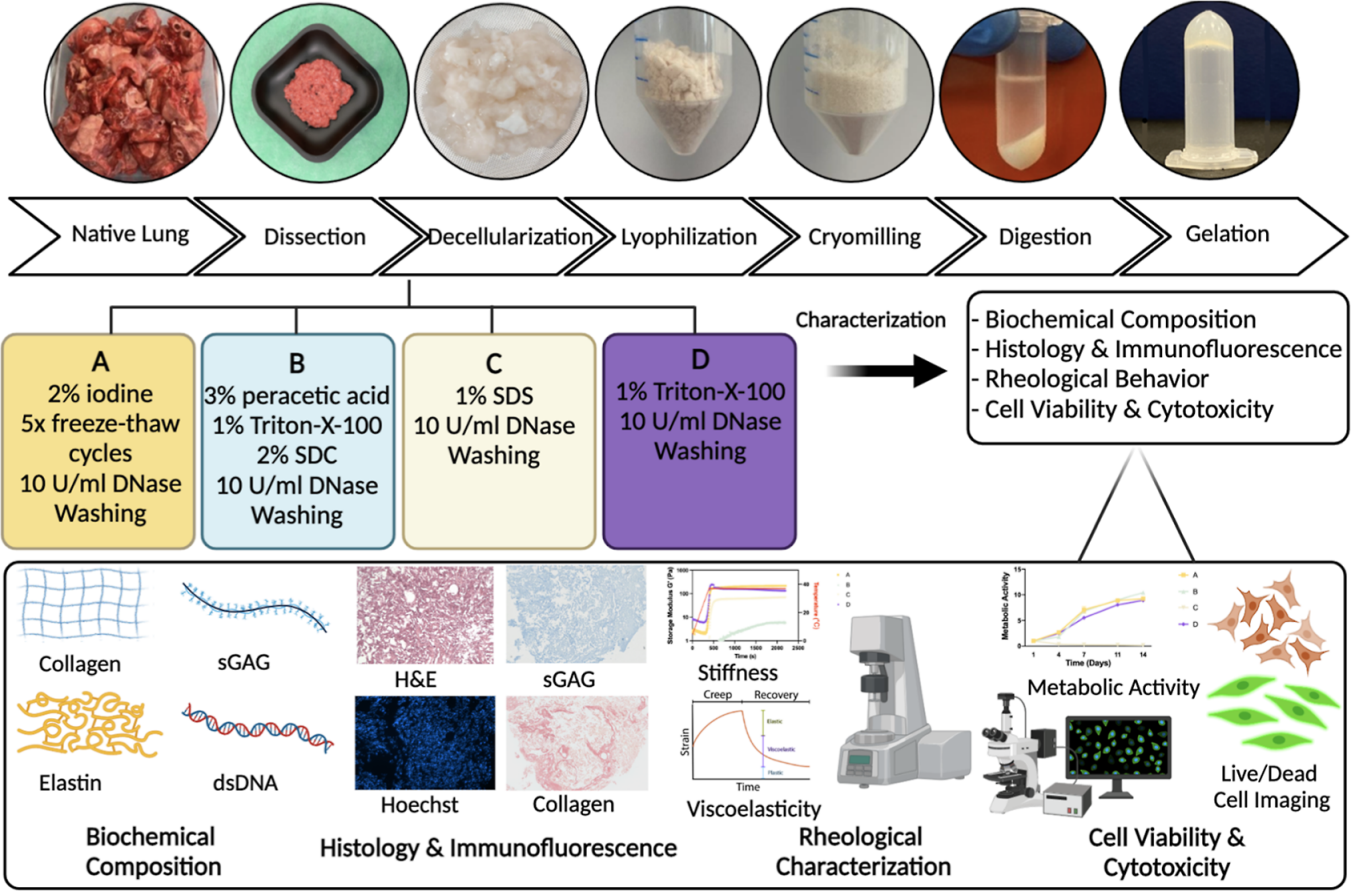
Schematic representation of the decellularization process and experimental characterizations.

We evaluated the impact of four decellularization methods on biochemical composition and mechanical characteristics of bovine lung dECM. Main steps of the process are briefly summarized in Figure 1 and involve dissection of lung tissues into small pieces, followed by one of the designated decellularization methods, lyophilization of decellularized tissues, cryomilling, solubilization of dECM by pepsin digestion, neutralization, and buffering to physiological conditions and thermal gelation to form lung dECM hydrogels (Figure 1). The effectiveness of decellularization methods of bovine lung tissues was first observed visually that all decellularized tissues lost their native pinkish color and turned into white, less rigid tissue pieces which indicates clearance of cellular material (Figure S1).

**Figure 2:**
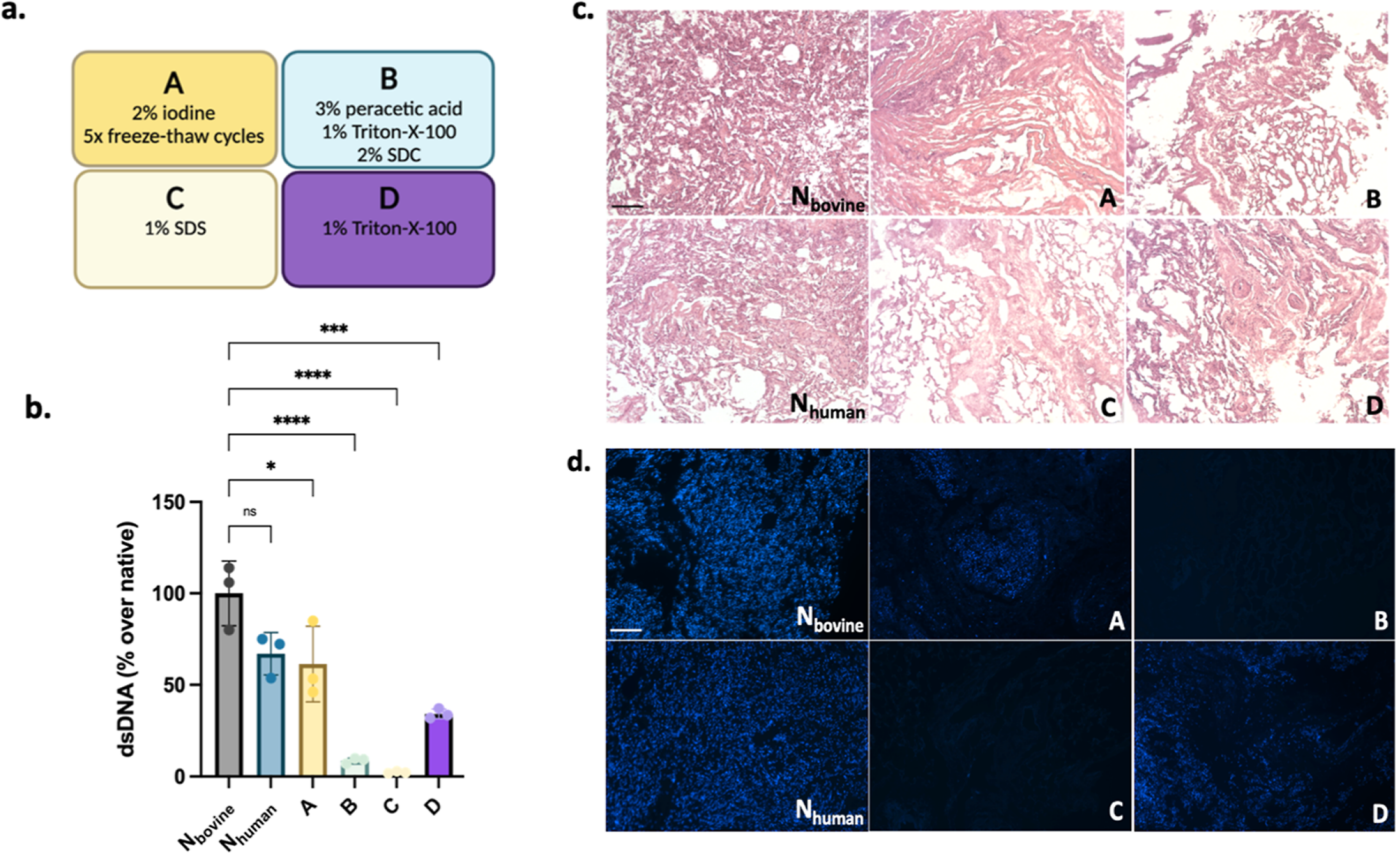
Evaluation of decellularization efficiency in dECM tissues. (a) Brief summary of decellularization methods. (b) Quantification of dsDNA in native and dECM tissues, all samples were normalized to native bovine tissue. (c) Histological examination of tissues stained with haematoxylin and eosin (scale bar: 100 μm). (d) Hoechst staining of native and dECM tissues (scale bar: 100 μm). N_bovine_ represents native bovine lung, and N_human_ represents native human lung. Error bars represent sd (ns, no significance, *p < 0.05, **p < 0.01, ***p < 0.001, ****p < 0.0001).

For quantified validation of decellularization, DNA content was determined in native and decellularized lung tissues (Figure 2a,b). In all biochemical characterizations throughout the study, native bovine tissues were compared to native human lung tissues to assess compositional differences between the two species and evaluate the use of bovine donors for tissue engineering applications to model human diseases. DNA content of native bovine and human tissues were found to be comparable (Figure 2b). On the other hand, all decellularization methods (A–D) in bovine tissue yielded significantly decreased dsDNA content compared to native bovine lung. Among the methods, B and C revealed the most effective removal of cellular DNA, whereas lung tissues treated with methods A and D showed some residual DNA (Figure 2b). Staining of native and decellularized tissues by the H&E and Hoechst nuclear dye demonstrated consistence with the DNA content (Figure 2c,d). Interestingly, when we performed DNA quantification after pepsin digestion, DNA content in decellularized lung tissues for all methods were further reduced (Figure S2). The effect was particularly pronounced for the freeze-thaw method A, where DNA removal was drastically improved.

### 3.2 Biochemical Characterization of the Decellularized Tissues

**Figure 3:**
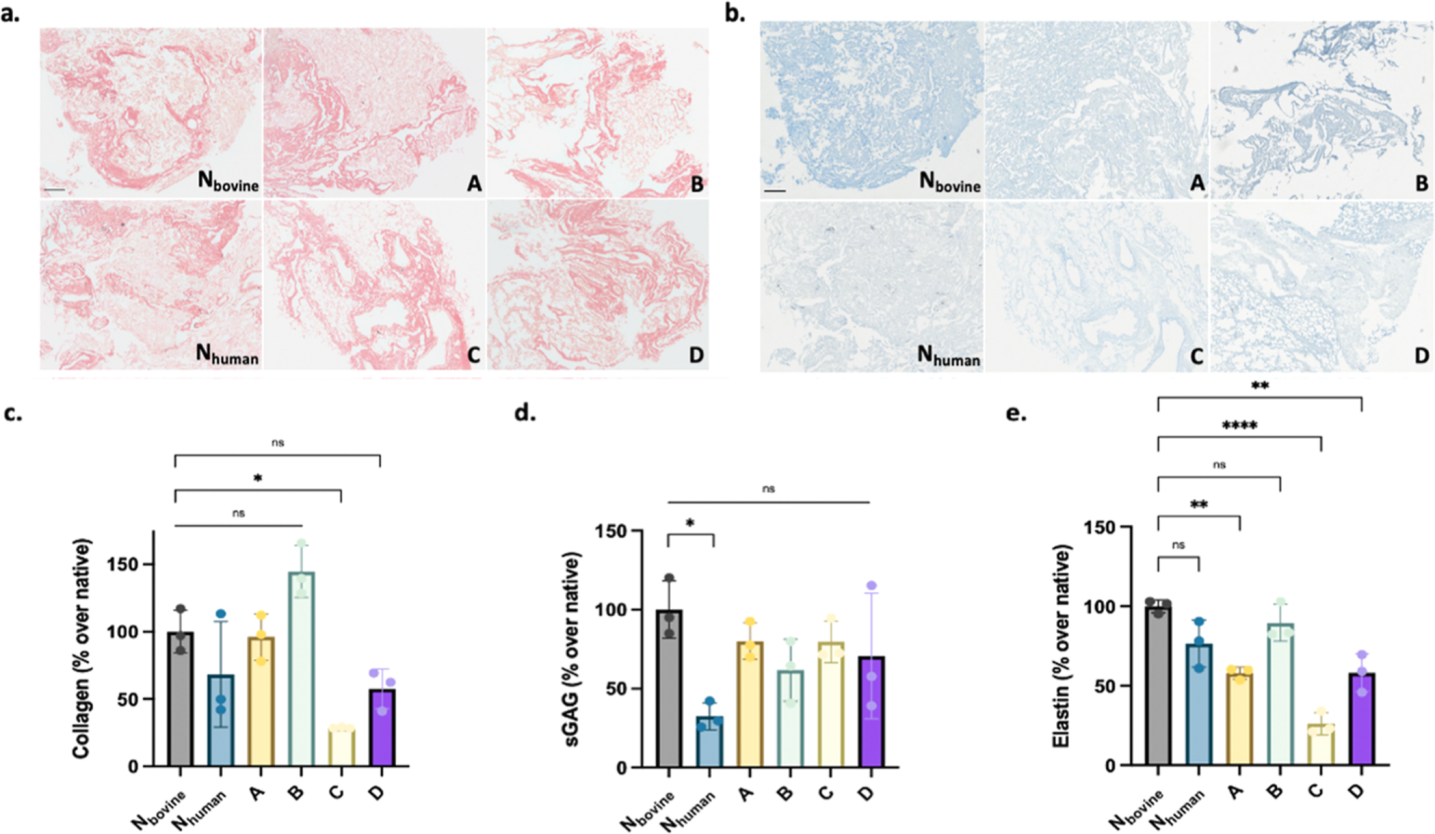
Histological and biochemical analysis of native and decellularized lung tissues. (a) Sirius red staining for collagen. (b) Alcian blue (sGAG) staining of native and decellularized lung tissues (scale bar: 100 μm). (c) Collagen quantification, (d) sGAG quantification, and (e) elastin quantification of native and decellularized lung tissues. All samples were normalized to native bovine. N_bovine_ represents native bovine lung, and N_human_ represents native human lung. Error bars represent sd (ns, no significance, *p < 0.05, **p < 0.01, ***p < 0.001, ****p < 0.0001).

Apart from the characterization of the remaining DNA in decellularized lung tissues, identifying the biochemical composition of the retained ECM is critical to assess how well they represent the native tissue ECM. Therefore, to determine the changes in the ECM composition due to decellularization, histological staining and colorimetric quantification of collagen, sGAG, and elastin were performed for native bovine (N_bovine_) and human (N_human_) lung tissues along with the decellularized bovine lung tissues (A, B, C, and D) (Figure 3).

To show retained collagen in the decellularized tissues, we performed histological Sirius red staining and quantification with Sircol assay (Figure 3a,c). Collagen content of the native bovine and human lungs was comparable. Although human lung samples revealed significant batch-to-batch variability, bovine donors were rather consistent in composition (Figure 3c). Decellularization of bovine tissues with methods A, B, and D did not yield any loss in collagen content, whereas method C (SDS treatment) revealed a significant decrease (Figure 3c). We then characterized the effect of decellularization on sGAG content in native and dECM tissues (Figure 3b,d). Native bovine lungs had remarkably higher sGAG content compared to human lungs. On the other hand, decellularization of bovine lungs did not cause any sGAG loss regardless of the pursued method (A–D) (Figure 3b,d). Elastin quantification was performed with Fastin assay and showed that elastin content of human and bovine lungs was not significantly different (Figure 3e). Upon decellularization, method B demonstrated most effective retention of elastin. Methods A and D led to preservation of over 60% of soluble elastin compared to native bovine tissue; however, method C resulted in the loss of approximately 75% of native elastin content (Figure 3e). Overall, crucial ECM components collagen, sGAGs, and elastin were highly preserved in three of four proposed decellularization methods. We then continued with assessing how mechanical properties of constituted lung dECM hydrogels are altered by the given differences in their biochemical composition.

### 3.3 Gelation of Lung dECM and Mechanical Characterization

Lung dECM powders derived from methods A–D were digested with pepsin to be able to achieve reconstitution via thermal crosslinking and several parameters including solvent acidity, dECM concentration, digestion time, soluble content, and storage conditions were screened for optimal gelation (Figure S3).

**Figure 4:**
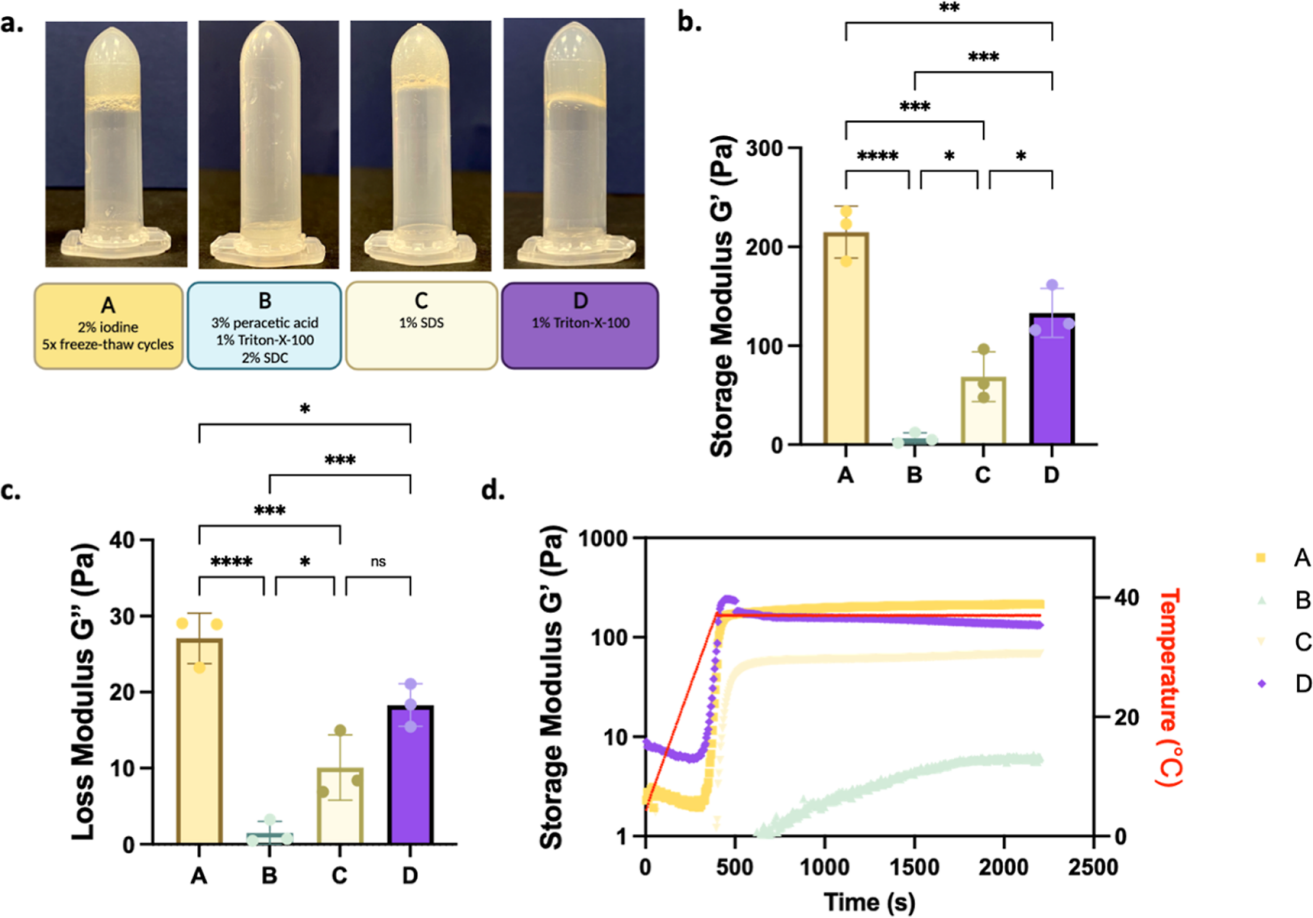
Gelation potential of dECM solutions and mechanical characterizations with oscillatory shear rheology. (a) Representative images of dECM solutions to gel transition after thermal crosslinking. (b) Rheological properties of dECM solutions, storage modulus (*G*). (c) Loss modulus of hydrogels (*G*″). (d) Temperature and time sweep showing gelation kinetics for different dECM samples. Error bars represent sd (ns, no significance, *p < 0.05, **p < 0.01, ***p < 0.001, ****p < 0.0001).

Optimal digestion time was determined as 48 h. Concentration of dECM in the digest buffer was varied as 10, 15, and 20 mg/mL for each method. 10 mg/mL digests were inconsistent and unsatisfactory in thermal gelation capabilities of the reconstituted dECM after neutralization and buffering to physiological conditions. On the other hand, 20 mg/mL samples demonstrated inhomogeneous digestion or premature gelation prior to neutralization and incubation at 37 °C which led to a poor handling of the digest (Figure S4a). Therefore, we have chosen 15 mg/mL to proceed with all the mechanical characterizations and cell growth studies for all methods. Furthermore, we have evaluated the formation of constituted hydrogels for both total and soluble digests (Figure S4b). Soluble digests outperformed total digests and yielded in more homogeneous, consistent, and transparent hydrogels after neutralization and buffering followed by thermal gelation. Therefore, the gelation confirmation with tube inversion assay was performed for the hydrogels of 15 mg/mL soluble digests derived from methods A–D (Figure 4a).

Methods A, C, and D demonstrated very homogeneous and successful thermal gelation; however, method B yielded very poor gelation and failed the inversion test. Rheological properties of dECM gel precursor solutions for each decellularization method were assessed using temperature ramping oscillatory rheology. Three decellularization methods (A, C, and D) exhibited gel-like characteristics because their storage modulus (*G*′) was significantly higher than the loss modulus (*G*″), whereas method B demonstrated the lack of gelation (Figure 4b–d). Lung dECM hydrogels derived from method A were significantly stiffer than hydrogels from methods C and D. We also determined that all dECM precursors start to show gelation properties when the temperature exceeds 30 °C and exhibit complete gelation in total 500 s into the temperature ramp (Figure 4d). Regarding the inability of gelation for method B, we questioned the effect of thorough mincing procedure which we performed on the lung tissues prior to the decellularization process to ensure effective chemical treatment and removal of cellular content. When we proceeded with method B after manual dissection of lung tissue pieces but skipped rigorous mincing, method B digests showed ability for gelation (Figure S5). However, this further mincing to increase surface area was necessary to ensure successful decellularization and removal of cellular material from the tissues.

**Figure 5:**
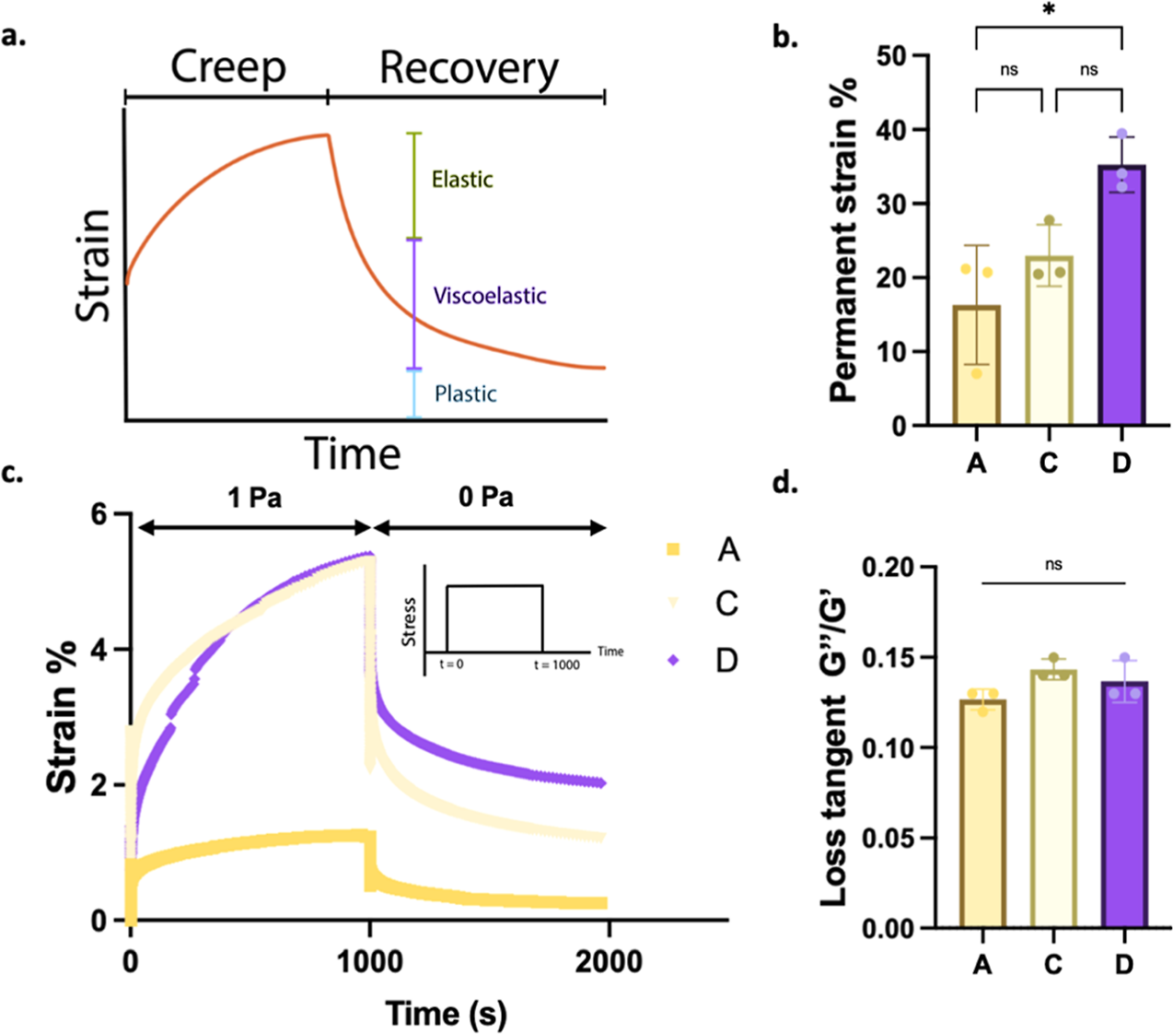
Viscoelasticity of dECM hydrogels. (a) Schematic representation of a creep curve. (b) Permanent strain preserved in dECM gels. (c) Creep recovery test. (d) Loss tangent. Error bars represent sd (ns, no significance, *p < 0.05).

In this study, we also performed creep and recovery tests to measure the response of dECM hydrogels obtained from four different decellularization methods and to demonstrate how they behave under a certain stress (Figure 5a). Hydrogels obtained by methods A, C, and D showed a degree of plasticity between 15 and 35% under a 1 Pa creep stress similar to collagen gels;^41^ however, plasticity data for dECM solution from method B could not be obtained due to improperly weak gelation and complete deformation under 1 Pa stress (Figure 5b–d). A-dECM gel demonstrated significantly lower plasticity, in other words, slower relaxation, compared to D-dECM gel, whereas degree of plasticity was similar between C-dECM and D-dECM gels even though their storage moduli were different (Figure 5b). Furthermore, viscosity of the dECM gels indicated by the loss tangent was also similar for three decellularization methods (Figure 5d). Briefly, these results indicate that different decellularization techniques on native bovine lung tissue markedly affect the viscoelastic properties of dECM gels.

### 3.4 Cytocompatibility and Cellular Growth in Lung dECM Hydrogels

**Figure 6:**
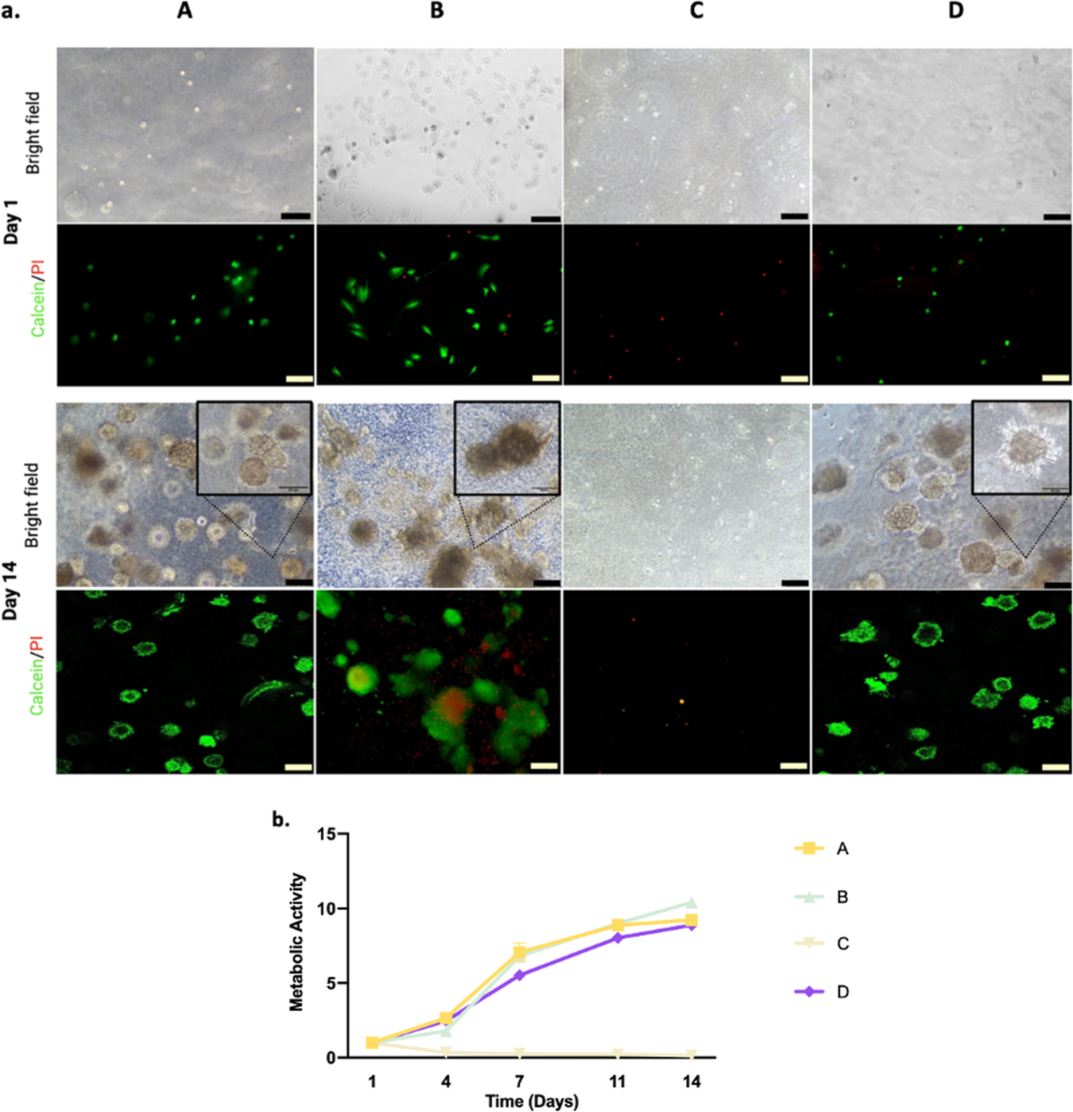
Cell viability and cytocompatibility assessments. (a) Representative images of cells stained for calcein-AM (green) and PI (red) on days 1 and 14 (scale bars: 100 μm). Zoomed in bright field images (scale bars: 50 μm) to represent clump morphologies. (b) Metabolic activity results from days 4, 7, 11, and 14 were normalized to day 1 for each method.

Lung adenocarcinoma (A549) cells were encapsulated in lung dECM hydrogels derived from methods A–D, cultured for 2 weeks, and monitored for cellular growth and morphology (Figure 6). The effect of varying biochemical composition, stiffness and viscoelasticity of different dECM hydrogels on cellular behavior was observed. Live-dead staining was performed with calcein-AM and PI to assess effects on cell viability at day 1 and day 14. Methods A, B, and D hydrogels efficiently supported cell viability at day 1 and showed good cytocompatibility. However, method C hydrogels exhibited a drastic loss of cell viability and lack of further growth (Figure 6a,b). Extra extensive washes were implemented into method C decellularization to assess whether cytocompatibility could be improved (Figure S6); however, even though cell density was increased to 1 million cells mL^–1^, the cytotoxic effect was still observed in C-dECM hydrogels (Figure S7). Cell growth was monitored at designated time points with a metabolic activity assay which showed constant increase for methods A, B, and D, indicating that these lung dECM hydrogels provided a supportive microenvironment for proliferation of lung cancer cells (Figure 6b). Cells exhibited growth in clump forms where invasive outgrowth was observed in A- and D-dECM hydrogels (Figure 6a). In terms of cellular morphology, there was no difference between A- and D-dECM even though stiffness and viscoelasticity of the hydrogels were significantly different (Figures 4–6). We also modulated the stiffness of A-dECM hydrogels via decreasing the ligand content that would yield a similar storage modulus to that of D-dECM hydrogels and analyzed the effect of stiffness on cellular growth and morphology (Figures S8 and S9). However, viability and morphology of A549 cells in soft versus stiff A-dECM hydrogels were similar (Figure S9).

On the other hand, because B-dECM resulted in weak gelation capacity and poor mechanical properties, cells showed monolayered adherent morphology along with the cell clumps in these hydrogels. Moreover, cell clumps formed in mechanically unstable B-dECM hydrogels were observed to be bigger than in A- and D-dECM and some of them showed core necrosis (Figure 6a).

**Figure 7:**
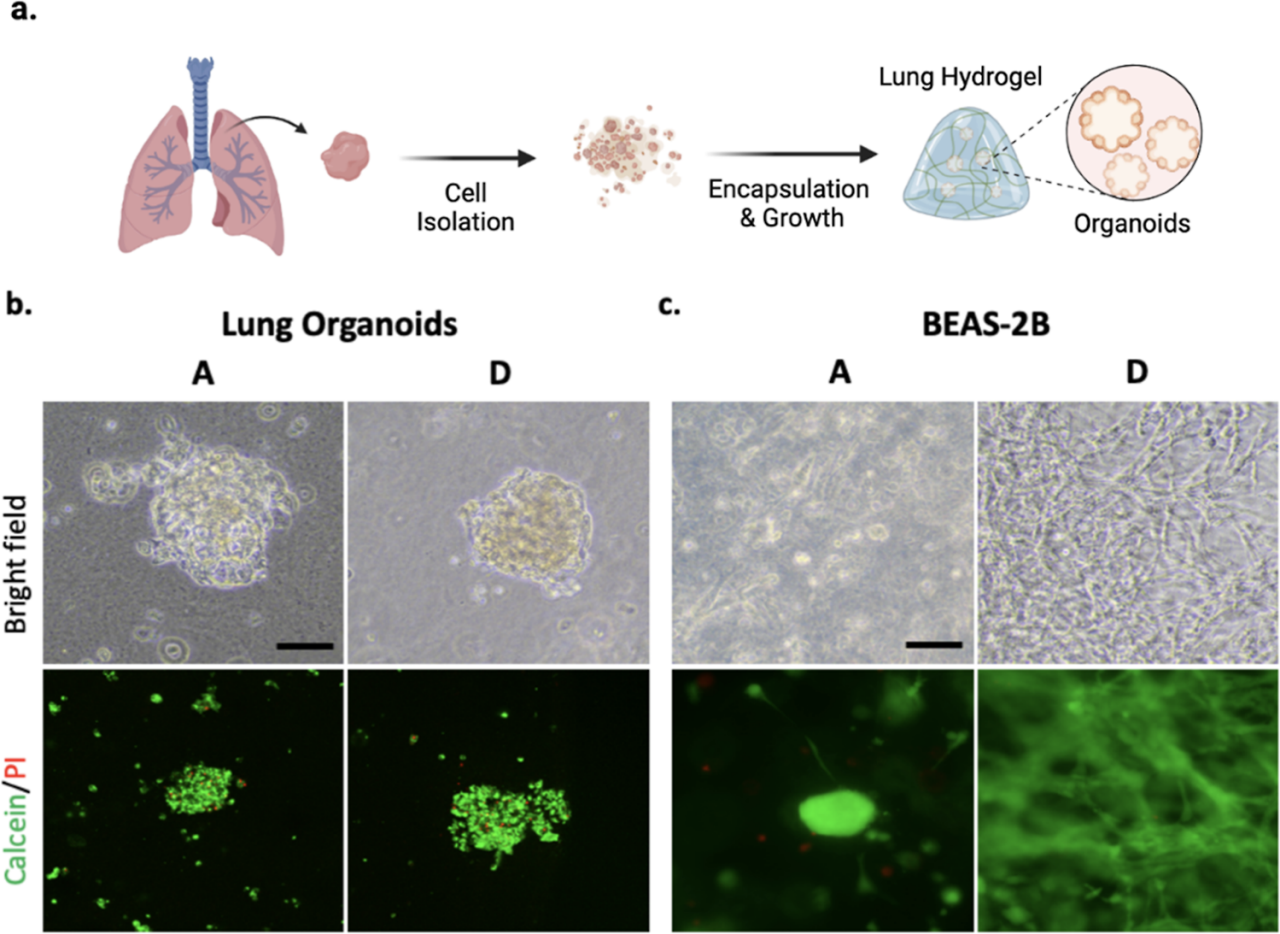
Cell viability assessment of non-tumorigenic human lung cells. (a) Schematic representation of human lung tissue collection, isolation of pulmonary epithelium, and culturing of lung organoids in lung dECM hydrogels. (b) Representative images of lung organoids encapsulated in A-dECM and D-dECM hydrogels and stained for calcein-AM (green) and PI (red) on day 10 (scale bar: 70 μm). (c) Representative images of BEAS-2B cell line encapsulated in A-dECM and D-dECM hydrogels and stained for calcein-AM (green) and PI (red) on day 10 (scale bar: 70 μm).

Next, we wanted to test the cytocompatibility of dECM hydrogels on non-tumorigenic cells. Patient-derived lung organoids were generated through isolation of pulmonary epithelium from non-tumorous human lung parenchyma taken from lung cancer patients who underwent lobectomy procedure as part of their treatment (Figure 7a). Serially passaged organoids were then encapsulated in either A-dECM or D-dECM hydrogels. Live-dead assay was performed at day 10 which showed that both A-dECM and D-dECM hydrogels provided a supportive niche for patient-derived lung organoids with optimal viability (Figure 7b). Bright field microscopy images revealed that lung organoid morphology was preserved in dECM hydrogels (Figure 7b). We also investigated whether A-dECM and D-dECM hydrogels maintain cellular viability of a bronchial epithelial cell line, BEAS-2B. Live-dead assay revealed that both dECM hydrogels showed cytocompatibility as most cells were stained only for calcein-AM (Figure 7c). Interestingly, cellular morphology of BEAS-2B cells encapsulated in A-dECM and D-dECM was remarkably different. In A-dECM hydrogels, cells tended to grow mostly as clusters; however, in D-dECM hydrogels, cells adapted a spread, mesenchymal-like morphology upon the differences in biochemical composition and mechanical properties of these hydrogels.

## 4 Discussion

Organ-derived hydrogels offer great promise because these biomaterials can be used to recapitulate the native tissue ECM that supports organotypic cellular function. Decellularized lung ECM has been used in tissue engineering approaches; however, a thorough characterization of the effect of different decellularization protocols on ECM composition and mechanical properties of reconstituted scaffolds, particularly stiffness and viscoelasticity, presents a gap in the field.^42^ In this study, we established bovine lung decellularization for the first time and pursued four different protocols which were evaluated in terms of retention of native ECM components and mechanical properties of the resulting dECM hydrogels (Supporting Information Table). Our protocol choices (A: freeze-thaw cycles; B: peracetic acid, Triton-X-100, SDC; C: SDS; D: Triton-X-100) aimed to cover the widely followed methods for organ decellularization with different strengths and weaknesses.^19,24,37,43,44^ All methods were supported by DNase treatment for further fragmentation of the residual DNA that could cause immunological response and decrease cell viability after encapsulation.^14^ All methods effectively removed cellular content after decellularization steps, remaining trace amounts of DNA only in methods A and D, which was further decreased after pepsin digestion (Figures 2 and S2). A small amount of residual DNA has been reported for many commercially available ECM products, suggesting that it does not lead to adverse host response or affect the potential uses of dECM gels in tissue engineering applications.^45^

ECM is composed of many protein and polysaccharide macromolecules including collagens, elastins, proteoglycans; thus, it is crucial to demonstrate the retention of native ECM proteins which are important for cellular signaling.^46^ Collagens are the most abundant matrix proteins among the macromolecules constituting the tissue matrices. Deposition, degradation, or post-translational modifications of collagen by the cells alter the mechanical strength of tissues and are linked to pathological conditions.^1^ According to our results, collagens were retained in three of four decellularization methods, except method C (Figure 3) which is consistent with some previous studies on lung tissue decellularization.^13^ In contrast, other studies showed preservation of collagen with SDS-based decellularization.^14,37^ Differences in findings could be attributed to changes in tissue type, species, concentration of SDS, and treatment duration. Even though SDS is a widely pursued reagent in the decellularization process, considering its effectiveness on DNA removal, it is critical to determine the optimal conditions to minimize loss of ECM proteins and cytotoxicity. We also observed a slight increase in collagen content in tissues decellularized with method B compared to the native tissue (Figure 3c). We propose that it could be related with the tissue shrinkage during peracetic acid treatment where such shrinkage leads to a denser matrix after decellularization, changing the amount of collagen per weighted tissue.^47^

sGAGs have crucial biological functions such as regulating cell behavior by direct interaction with cell surface receptors. In the ECM, they can sequester bioactive ligands and act as a reservoir for growth factors that play a significant role in cell growth and function.^48^ In literature, various lung decellularization protocols were shown to cause a loss of GAG content, which has been proposed to hinder ligand-mediated cellular signaling within decellularized lung hydrogels.^48^ Therefore, re-addition of GAGs into the scaffolds to compensate for the decellularization-mediated loss was shown to have a differential effect on cell growth.^48^ Moreover, remaining sGAGs can affect the mechanical properties of gels derived from decellularized tissues because these molecules also promote water retention which in turn modulate viscosity of the dECM gels.^49^ All decellularization methods in this study were able to retain sGAG molecules, suggesting that all methods are ideal to maintain native sGAG content in dECM gels. Together with this, we observed that human lungs derived from patients had significantly lower amount of sGAGs compared to bovine and their collagen content was very variable (Figure 3). Because the composition of normal lung parenchyma could be affected by lung-related diseases or age, potential sGAG and collagen alterations in human tissues point out an advantage of using non-human-derived tissues for engineering purposes.^48^ Therefore, in this study, bovine lung is introduced as a more stable, reproducible natural ECM source for modeling the human lung microenvironment.

Elastin is another critical component of the lung ECM that is responsible for the elasticity of the organ both in vasculature and parenchyma.^50^ It has been previously shown that decellularization methods that lead to elastin retention enable the formation of scaffolds mechanically similar to native lung in terms of elastic behavior and tensile strength.^13^ Elastin preservation in methods A, B, and D (Figure 3) and loss upon SDS-based methods were compatible with the literature.^13,51^ Collectively, these results indicate that the decellularization approach is an important factor determining the remnant ECM proteins that play crucial roles in physical properties of reconstituted scaffolds and cell-ECM interactions.

Pepsin digestion in dilute acids has been commonly used to solubilize dECM materials by cleaving the telopeptide bonds of collagens that leads to free collagen chains to self-assemble after pH neutralization under optimal temperature conditions.^52^ A recent study suggests that porcine dECM pre-gel solutions obtained by 24 h or shorter pepsin digestion times lead to mechanically more stable gels due to the physical architecture of the dECM.^52^ Contrarily, we observed that pepsin digestion times shorter than 24 h failed the complete solubilization of bovine lung dECM powder, resulting in poor and heterogeneous gelation. This difference may rely on the complexity of the dECM because there are various factors defining an effective solubilization process, such as enzyme to substrate ratio, pH, and temperature. Even decellularization methods could affect the pepsin digestion protocol; thus, it is important to optimize the digestion for specific experimental conditions to obtain homogeneous and biocompatible dECM hydrogels.

Mechanical properties of the 3D microenvironment are extremely important for cell culture studies because the growth kinetics of the cells depend on mechanical characteristics of the ECM as well as the biochemical composition.^1,6,12^ Here, we showed that different decellularization techniques had distinct effects on gelation and the final stiffness and viscoelasticity of dECM hydrogels. Our results indicated that, decellularization method A which is based on physical disruption of cellular components of native tissue by freeze-thaw cycles resulted in more stable and stiffer hydrogels compared to other methods (Figure 4). Together with this, we revealed that stiffness of A-dECM hydrogels can be manipulated by varying the ligand concentration and can be coupled to the stiffness of D-dECM hydrogels (Figure S8). Importantly, both stiff and soft A-dECM hydrogels supported cellular viability, revealing that modulation of mechanical properties of dECM-based hydrogels were possible without compromising cytocompatibility (Figure S9).

Additionally, gelation profiles and storage moduli obtained by rheology were comparable to reported dECM hydrogels derived from other tissues.^19^ A recent study comparing the impact of decellularization methods on corneas demonstrated that mechanical properties and cytocompatibility of dECM hydrogels produced by the freeze-thaw method showed great promise arguing that chemical disruption of ECM proteins using common detergents negatively affects dECM hydrogel mechanics.^14^ Even though dECM hydrogels obtained here do not match the stiffness range of the healthy lung tissue (1–5 kPa),^39^ the freeze-thaw method presents a clear improvement in the mechanical stability and stiffness of dECM gels. Tissue engineering offers strategies to mechanically reinforce dECM hydrogels to desired ranges specific to application such as combination of dECM hydrogels with biopolymers that are tunable in terms of physio-chemical properties.^53^

Viscoelasticity is a characteristic of living tissues and ECMs that display both viscous and elastic responses to mechanical deformation. Viscoelastic materials can be deformed or, in other words, creep, in response to a force. When the external force is removed, materials undergo “recovery” or “stress relaxation” in a time-dependent manner.^12^ Materials that show high degree of plasticity can sustain deformation permanently, whereas viscoelastic materials are able to sustain deformation semi-permanently. Recent studies revealed that ECM viscoelasticity plays key roles in cell proliferation, morphology, and differentiation.^12,54^ Here, we showed that lung dECM hydrogels are stress-relaxing materials similar to native fibrin, collagen, or reconstituted basement membrane.^54^ Our results show that D-dECM hydrogels demonstrate faster recovery compared to A-dECM hydrogels, indicating that in fact, decellularization methods used here had an impact on creep response of resulting dECM gels. To our knowledge, this is the first report characterizing viscoelastic behavior of lung dECM hydrogels which allows a study of the effect of time-dependent mechanics of dECM on cellular behavior. Following biochemical and mechanical characterizations, we assessed cytocompatibility, cellular growth, and morphology in lung dECM hydrogels (Figures 6 and 7). A-, B-, and D-dECM were highly supportive of cell viability and growth of the A549 cell line, whereas SDS-based C-dECM resulted in immediate and complete loss of cell viability. The negative effect of SDS-based decellularization on cell viability has been reported and remnant detergent was shown as a possible reason.^14^ Cell viability with this method did not improve despite implementation of extra extensive washes (Figure S6) to ensure removal of detergent in this study. During the decellularization process, SDS might harm the native lung matrix composition by damaging the essential matrix proteins due to its strongly anionic nature which could potentially harm cytocompatibility. Contrarily, SDS-based dECM hydrogels have been shown for various other tissues with good cell viability.^44^ B-dECM hydrogels have poor gelation capacity; however, apart from mechanical instability, method B showed very good cytocompatibility (Figure 6). Cell growth and morphology in A- and D-dECM were comparable despite the differences in their mechanical properties. A-dECM hydrogels were stiffer and stiffness is a well-established trigger of malignant cell growth.^5,7^ On the other hand, D-dECM hydrogels showed faster stress relaxation and higher plasticity which has been linked to altered cellular behavior such as malignant phenotype and migration.^55^ Different mechanical aspects could have been compensatory in the aforementioned methods to result in similar cellular growth.

Encapsulation of bronchial epithelial cells (BEAS-2B) and patient-derived lung organoids into A-dECM and D-dECM gels revealed optimal cytocompatibility of dECM hydrogels with non-tumorous cells as well. Interestingly, A-dECM hydrogels triggered mostly spheroidal growth of BEAS-2B cells, whereas cells in D-dECM hydrogels showed completely spread morphology (Figure 7). BEAS-2B cells were previously shown to have the capability of exhibiting mesenchymal characteristics and we showed that the morphology and behavior of these cells were greatly affected from the biophysical aspects of their microenvironment.^56^ Furthermore, ECM has been shown to be a decisive factor for the expansion of organoid cultures in terms of its effect on morphology and cellular differentiation.^57^ dECM hydrogels offer a promising and organotypic microenvironment with physiologically relevant biochemical and physical cues for growth of healthy pulmonary epithelium and patient-derived organoids. Overall, methods A and D showed great potential for the use of dECM hydrogels in disease modeling of the lung.

## 5 Conclusions

In conclusion, we established decellularization of bovine lungs and showed the impact of using different protocols on biochemical and mechanical properties of reconstituted dECM hydrogels. Bovine lung tissues demonstrated a good alternative to human lungs for use in tissue engineering with comparable or better ECM retention and much improved batch-to-batch variability. Mechanical inputs are crucial factors determining the cellular fate, and our study sheds light on how decellularization affects stiffness and viscoelasticity which is a step further toward building tissue models with faithful recapitulation of native tissue characteristics.

## Supporting Information Available

Visual assessment of decellularization, dsDNA quantification after pepsin digestion, optimization parameters of the enzymatic digestion and storage, premature gelation of digests, effect of tissue sizes on storage modulus of dECM, storage modulus of dECM hydrogels with varying ligand densities, lung cancer cells encapsulation in stiff and soft hydrogels, elimination of residual detergent with extra extensive wash, calcein-PI staining, and comparative summary table (PDF)

## Abbreviations

CHAPS: 3-[(3-cholamidopropyl)dimethylammonio]-1-propanesulfonate
COPD: chronic obstructive pulmonary disease
dECM: decellularized extracellular matrix
ECM: extracellular matrix
PBS: phosphate-buffered saline
PI: propidium iodide
SDC: sodium deoxycholate
SDS: sodium dodecyl sulfate
sGAG: sulfated glycosaminoglycan

## References

[ref1] Cox T. The matrix in cancer. Nat. Rev. Cancer 2021, 21, 217–238. 10.1038/s41568-020-00329-7. 33589810

[ref2] Muncie J. M.; Weaver V. M. The Physical and Biochemical Properties of the Extracellular Matrix Regulate Cell Fate. Curr. Top. Dev. Biol. 2018, 130, 1–37. 10.1016/bs.ctdb.2018.02.002. 29853174 PMC6586474

[ref3] Frantz C.; Stewart K. M.; Weaver V. M. The extracellular matrix at a glance. J. Cell Sci. 2010, 123, 4195–4200. 10.1242/jcs.023820. 21123617 PMC2995612

[ref4] Leiva O.; Ng S. K.; Chitalia S.; Balduini A.; Matsuura S.; Ravid K. The role of the extracellular matrix in primary myelofibrosis. Blood Cancer J. 2017, 7, e525 10.1038/bcj.2017.6. 28157219 PMC5386340

[ref5] Stowers R. S.; Shcherbina A.; Israeli J.; Gruber J. J.; Chang J. L.; Nam S.; Rabiee A.; Teruel M. N.; Snyder M. P.; Kundaje A.; Chaudhuri O. Matrix stiffness induces a tumorigenic phenotype in mammary epithelium through changes in chromatin accessibility. Nat. Biomed. Eng. 2019, 3, 1009–1019. 10.1038/s41551-019-0420-5. 31285581 PMC6899165

[ref6] Vining K. H.; Mooney D. J. Mechanical forces direct stem cell behaviour in development and regeneration. Nat. Rev. Mol. Cell Biol. 2017, 18, 728–742. 10.1038/nrm.2017.108. 29115301 PMC5803560

[ref7] Wei J.; Yao J.; Yan M.; Xie Y.; Liu P.; Mao Y.; Li X. The role of matrix stiffness in cancer stromal cell fate and targeting therapeutic strategies. Acta Biomater. 2022, 150, 34–47. 10.1016/j.actbio.2022.08.005. 35948177

[ref8] Wang C.; Sinha S.; Jiang X. Y.; Murphy L.; Fitch S.; Wilson C.; Grant G.; Yang F. Matrix Stiffness Modulates Patient-Derived Glioblastoma Cell Fates in Three-Dimensional Hydrogels. Tissue Eng., Part A 2021, 27, 390–401. 10.1089/ten.TEA.2020.0110. 32731804 PMC7984937

[ref9] Zhu X. L.; Li Y.; Yang Y.; He Y. T.; Gao M. Y.; Peng W. L.; Wu Q.; Zhang G. Y.; Zhou Y. Y.; Chen F.; Bao J.; Li W. M. Ordered micropattern arrays fabricated by lung-derived dECM hydrogels for chemotherapeutic drug screening. Mater. Today Bio 2022, 15, 100274 10.1016/j.mtbio.2022.100274. PMC9114688 35601895

[ref10] Humphrey J.; Dufresne E.; Schwartz M. Mechanotransduction and extracellular matrix homeostasis. Nat. Rev. Mol. Cell Biol. 2014, 15, 802–812. 10.1038/nrm3896. 25355505 PMC4513363

[ref11] Chaudhuri O. Viscoelastic hydrogels for 3D cell culture. Biomater. Sci. 2017, 5, 1480–1490. 10.1039/c7bm00261k. 28584885

[ref12] Chaudhuri O.; Cooper-White J.; Janmey P. A.; Mooney D. J.; Shenoy V. B. Effects of extracellular matrix viscoelasticity on cellular behaviour. Nature 2020, 584, 535–546. 10.1038/s41586-020-2612-2. 32848221 PMC7676152

[ref13] Petersen T. H.; Calle E. A.; Colehour M. B.; Niklason L. E. Matrix composition and mechanics of decellularized lung scaffolds. Cells Tissues Organs 2012, 195, 222–231. 10.1159/000324896. 21502745 PMC3696368

[ref14] Fernández-Pérez J.; Ahearne M. The impact of decellularization methods on extracellular matrix derived hydrogels. Sci. Rep. 2019, 9, 14933 10.1038/s41598-019-49575-2. 31624357 PMC6797749

[ref15] Rahman S.; Griffin M.; Naik A.; Szarko M.; Butler P. E. M. Optimising the decellularization of human elastic cartilage with trypsin for future use in ear reconstruction. Sci. Rep. 2018, 8, 3097 10.1038/s41598-018-20592-x. 29449572 PMC5814427

[ref16] Hashimoto Y.; Hattori S.; Sasaki S.; Honda T.; Kimura T.; Funamoto S.; Kobayashi H.; Kishida A. Ultrastructural analysis of the decellularized cornea after interlamellar keratoplasty and microkeratome-assisted anterior lamellar keratoplasty in a rabbit model. Sci. Rep. 2016, 6, 27734 10.1038/srep27734. 27291975 PMC4904214

[ref17] Seo Y.; Jung Y.; Kim S. H. Decellularized heart ECM hydrogel using supercritical carbon dioxide for improved angiogenesis. Acta Biomater. 2018, 67, 270–281. 10.1016/j.actbio.2017.11.046. 29223704

[ref18] Singelyn J. M.; Christman K. L. Modulation of material properties of a decellularized myocardial matrix scaffold. Macromol. Biosci. 2011, 11, 731–738. 10.1002/mabi.201000423. 21322109 PMC3280095

[ref19] Pouliot R. A.; Link P. A.; Mikhaiel N. S.; Schneck M. B.; Valentine M. S.; Kamga Gninzeko F. J.; Herbert J. A.; Sakagami M.; Heise R. L. Development and characterization of a naturally derived lung extracellular matrix hydrogel. J. Biomed. Mater. Res., Part A 2016, 104, 1922–1935. 10.1002/jbm.a.35726. PMC7962169 27012815

[ref20] Sengyoku H.; Tsuchiya T.; Obata T.; Doi R.; Hashimoto Y.; Ishii M.; Sakai H.; Matsuo N.; Taniguchi D.; Suematsu T.; Lawn M.; Matsumoto K.; Miyazaki T.; Nagayasu T. Sodium hydroxide based non-detergent decellularizing solution for rat lung. Organogenesis 2018, 14, 94–106. 10.1080/15476278.2018.1462432. 29889592 PMC6150056

[ref21] Petersen T. H.; Calle E. A.; Zhao L.; Lee E. J.; Gui L.; Raredon M. B.; Gavrilov K.; Yi T.; Zhuang Z. W.; Breuer C.; Herzog E.; Niklason L. E. Tissue-engineered lungs for in vivo implantation. Science 2010, 329, 538–541. 10.1126/science.1189345. 20576850 PMC3640463

[ref22] Gilpin S. E.; Guyette J. P.; Gonzalez G.; Ren X.; Asara J. M.; Mathisen D. J.; Vacanti J. P.; Ott H. C. Perfusion decellularization of human and porcine lungs: bringing the matrix to clinical scale. J. Heart Lung Transplant. 2014, 33, 298–308. 10.1016/j.healun.2013.10.030. 24365767

[ref23] Loneker A. E.; Faulk D. M.; Hussey G. S.; D’Amore A.; Badylak S. F. Solubilized liver extracellular matrix maintains primary rat hepatocyte phenotypein-vitro. J. Biomed. Mater. Res., Part A 2016, 104, 957–965. 10.1002/jbm.a.35636. 26704367

[ref24] Beachley V.; Ma G.; Papadimitriou C.; Gibson M.; Corvelli M.; Elisseeff J. Extracellular matrix particle-glycosaminoglycan composite hydrogels for regenerative medicine applications. J. Biomed. Mater. Res., Part A 2018, 106, 147–159. 10.1002/jbm.a.36218. 28879659

[ref25] Lee J. S.; Shin J.; Park H.-M.; Kim Y.-G.; Kim B.-G.; Oh J.-W.; Cho S.-W. Liver extracellular matrix providing dual functions of two-dimensional substrate coating and three-dimensional injectable hydrogel platform for liver tissue engineering. Biomacromolecules 2014, 15, 206–218. 10.1021/bm4015039. 24350561

[ref26] Keane T. J.; Swinehart I. T.; Badylak S. F. Methods of tissue decellularization used for preparation of biologic scaffolds and in vivo relevance. Methods 2015, 84, 25–34. 10.1016/j.ymeth.2015.03.005. 25791470

[ref27] Poon C. J.; Pereira E. Cotta M. V. P. E.; Sinha S.; Palmer J. A.; Woods A. A.; Morrison W. A.; Abberton K. M. Preparation of an adipogenic hydrogel from subcutaneous adipose tissue. Acta Biomater. 2013, 9, 5609–5620. 10.1016/j.actbio.2012.11.003. 23142702

[ref28] Sood D.; Chwalek K.; Stuntz E.; Pouli D.; Du C.; Tang-Schomer M.; Georgakoudi I.; Black L. D. III; Kaplan D. L. Fetal brain extracellular matrix boosts neuronal network formation in 3D bioengineered model of cortical brain tissue. ACS Biomater. Sci. Eng. 2016, 2, 131–140. 10.1021/acsbiomaterials.5b00446. 29034320 PMC5636008

[ref29] Balestrini J. L.; Gard A. L.; Gerhold K. A.; Wilcox E. C.; Liu A.; Schwan J.; Le A. V.; Baevova P.; Dimitrievska S.; Zhao L.; Sundaram S.; Sun H.; Rittié L.; Dyal R.; Broekelmann T. J.; Mecham R. P.; Schwartz M. A.; Niklason L. E.; White E. S. Comparative biology of decellularized lung matrix: Implications of species mismatch in regenerative medicine. Biomaterials 2016, 102, 220–230. 10.1016/j.biomaterials.2016.06.025. 27344365 PMC4939101

[ref30] Saldin L. T.; Cramer M. C.; Velankar S. S.; White L. J.; Badylak S. F. Extracellular matrix hydrogels from decellularized tissues: Structure and function. Acta Biomater. 2017, 49, 1–15. 10.1016/j.actbio.2016.11.068. 27915024 PMC5253110

[ref31] Siegel R.; Miller K.; Fuchs H.; Jemal A. Cancer Statistics, 2021. Ca-Cancer J. Clin. 2021, 71, 7–33. 10.3322/caac.21654. 33433946

[ref32] Burney P. G. J.; Patel J.; Newson R.; Minelli C.; Naghavi M. Global and regional trends in COPD mortality, 1990-2010. Eur. Respir. J. 2015, 45, 1239–1247. 10.1183/09031936.00142414. 25837037 PMC4531307

[ref33] Huh D.; Matthews B. D.; Mammoto A.; Montoya-Zavala M.; Hsin H. Y.; Ingber D. E. Reconstituting organ-level lung functions on a chip. Science 2010, 328, 1662–1668. 10.1126/science.1188302. 20576885 PMC8335790

[ref34] Wagner D. E.; Bonenfant N. R.; Sokocevic D.; DeSarno M. J.; Borg Z. D.; Parsons C. S.; Brooks E. M.; Platz J. J.; Khalpey Z. I.; Hoganson D. M.; Deng B.; Lam Y. W.; Floreani R. A.; Ashikaga T.; Weiss D. J. Three-dimensional scaffolds of acellular human and porcine lungs for high throughput studies of lung disease and regeneration. Biomaterials 2014, 35, 2664–2679. 10.1016/j.biomaterials.2013.11.078. 24411675 PMC4215726

[ref35] Lin Y. M.; Zhang A.; Rippon H. J.; Bismarck A.; Bishop A. E. Tissue engineering of lung: the effect of extracellular matrix on the differentiation of embryonic stem cells to pneumocytes. Tissue Eng., Part A 2010, 16, 1515–1526. 10.1089/ten.tea.2009.0232. 20001250

[ref36] Daly A.; Wallis J.; Borg Z.; Bonvillain R.; Deng B.; Ballif B.; Jaworski D.; Allen G.; Weiss D. Initial Binding and Recellularization of Decellularized Mouse Lung Scaffolds with Bone Marrow-Derived Mesenchymal Stromal Cells. Tissue Eng., Part A 2012, 18, 1–16. 10.1089/ten.TEA.2011.0301. 21756220 PMC4066256

[ref37] O’Neill J. D.; Anfang R.; Anandappa A.; Costa J.; Javidfar J.; Wobma H. M.; Singh G.; Freytes D. O.; Bacchetta M. D.; Sonett J. R.; Vunjak-Novakovic G. Decellularization of human and porcine lung tissues for pulmonary tissue engineering. Ann. Thorac. Surg. 2013, 96, 1046–1056. 10.1016/j.athoracsur.2013.04.022. 23870827 PMC4033908

[ref38] Balestrini J. L.; Gard A. L.; Liu A.; Leiby K. L.; Schwan J.; Kunkemoeller B.; Calle E. A.; Sivarapatna A.; Lin T.; Dimitrievska S.; Cambpell S. G.; Niklason L. E. Production of decellularized porcine lung scaffolds for use in tissue engineering. Integr. Biol. 2015, 7, 1598–1610. 10.1039/c5ib00063g. PMC4666745 26426090

[ref39] Hinz B. Mechanical Aspects of Lung Fibrosis. Proc. Am. Thorac. Soc. 2012, 9, 137–147. 10.1513/pats.201202-017aw. 22802288

[ref40] Suki B.; Bates J. H. T. Lung tissue mechanics as an emergent phenomenon. J. Appl. Physiol. 2011, 110, 1111–1118. 10.1152/japplphysiol.01244.2010. 21212247 PMC3075131

[ref41] Nam S.; Lee J.; Brownfield D. G.; Chaudhuri O. Viscoplasticity Enables Mechanical Remodeling of Matrix by Cells. Biophys. J. 2016, 111, 2296–2308. 10.1016/j.bpj.2016.10.002. 27851951 PMC5113260

[ref42] Uriarte J. J.; Uhl F. E.; Rolandsson Enes S. E.; Pouliot R. A.; Weiss D. J. Lung bioengineering: advances and challenges in lung decellularization and recellularization. Curr. Opin. Organ Transplant. 2018, 23, 673–678. 10.1097/mot.0000000000000584. 30300330 PMC8669574

[ref43] Roth S. P.; Glauche S. M.; Plenge A.; Erbe I.; Heller S.; Burk J. Automated freeze-thaw cycles for decellularization of tendon tissue—a pilot study. BMC Biotechnol. 2017, 17, 13 10.1186/s12896-017-0329-6. 28193263 PMC5307874

[ref44] Hernandez M. J.; Yakutis G. E.; Zelus E. I.; Hill R. C.; Dzieciatkowska M.; Hansen K. C.; Christman K. L. Manufacturing considerations for producing and assessing decellularized extracellular matrix hydrogels. Methods 2020, 171, 20–27. 10.1016/j.ymeth.2019.09.015. 31546012

[ref45] Gilbert T. W.; Freund J. M.; Badylak S. F. Quantification of DNA in biologic scaffold materials. J. Surg. Res. 2009, 152, 135–139. 10.1016/j.jss.2008.02.013. 18619621 PMC2783373

[ref46] Crapo P. M.; Gilbert T. W.; Badylak S. F. An overview of tissue and whole organ decellularization processes. Biomaterials 2011, 32, 3233–3243. 10.1016/j.biomaterials.2011.01.057. 21296410 PMC3084613

[ref47] Aeberhard P.; Grognuz A.; Peneveyre C.; McCallin S.; Hirt-Burri N.; Antons J.; Pioletti D.; Raffoul W.; Applegate L. Efficient decellularization of equine tendon with preserved biomechanical properties and cytocompatibility for human tendon surgery indications. Artif. Organs 2020, 44, E161–E171. 10.1111/aor.13581. 31609006 PMC7154770

[ref48] Uhl F. E.; Zhang F.; Pouliot R. A.; Uriarte J. J.; Rolandsson Enes S.; Han X.; Ouyang Y.; Xia K.; Westergren-Thorsson G.; Malmström A.; Hallgren O.; Linhardt R. J.; Weiss D. J. Functional role of glycosaminoglycans in decellularized lung extracellular matrix. Acta Biomater. 2020, 102, 231–246. 10.1016/j.actbio.2019.11.029. 31751810 PMC8713186

[ref49] Sicari B. M.; Londono R.; Badylak S. F. Extracellular Matrix as a Bioscaffold for Tissue Engineering. In Tissue Engineering, 2nd ed.; Academic Press, 2014; pp 149−175. 10.1016/b978-0-12-420145-3.00005-5

[ref50] Balestrini J. L.; Niklason L. E. Extracellular matrix as a driver for lung regeneration. Ann. Biomed. Eng. 2015, 43, 568–576. 10.1007/s10439-014-1167-5. 25344351 PMC4380778

[ref51] Dabaghi M.; Saraei N.; Carpio M. B.; Nanduri V.; Ungureanu J.; Babi M.; Chandiramohan A.; Noble A.; Revill S. D.; Zhang B.; Ask K.; Kolb M.; Shargall Y.; Moran-Mirabal J.; Hirota J. A. A Robust Protocol for Decellularized Human Lung Bioink Generation Amenable to 2D and 3D Lung Cell Culture. Cells 2021, 10, 1538 10.3390/cells10061538. 34207111 PMC8234522

[ref52] Pouliot R. A.; Young B. M.; Link P. A.; Park H. E.; Kahn A. R.; Shankar K.; Schneck M. B.; Weiss D. J.; Heise R. L. Porcine Lung-Derived Extracellular Matrix Hydrogel Properties Are Dependent on Pepsin Digestion Time. Tissue Eng. C Methods 2020, 26, 332–346. 10.1089/ten.tec.2020.0042. PMC7310225 32390520

[ref53] Mendibil U.; Ruiz-Hernandez R.; Retegi-Carrion S.; Garcia-Urquia N.; Olalde-Graells B.; Abarrategi A. Tissue-Specific Decellularization Methods: Rationale and Strategies to Achieve Regenerative Compounds. Int. J. Mol. Sci. 2020, 21, 5447 10.3390/ijms21155447. 32751654 PMC7432490

[ref54] Chaudhuri O.; Gu L.; Klumpers D.; Darnell M.; Bencherif S. A.; Weaver J. C.; Huebsch N.; Lee H. P.; Lippens E.; Duda G. N.; Mooney D. J. Hydrogels with tunable stress relaxation regulate stem cell fate and activity. Nat. Mater. 2016, 15, 326–334. 10.1038/nmat4489. 26618884 PMC4767627

[ref55] Wisdom K. M.; Adebowale K.; Chang J.; Lee J. Y.; Nam S.; Desai R.; Rossen N. S.; Rafat M.; West R. B.; Hodgson L.; Chaudhuri O. Matrix mechanical plasticity regulates cancer cell migration through confining microenvironments. Nat. Commun. 2018, 9, 4144 10.1038/s41467-018-06641-z. 30297715 PMC6175826

[ref56] Han X.; Na T.; Wu T.; Yuan B. Z. Human lung epithelial BEAS-2B cells exhibit characteristics of mesenchymal stem cells. PLoS One 2020, 15, e0227174 10.1371/journal.pone.0227174. 31900469 PMC6941928

[ref57] Jeon E. Y.; Sorrells L.; Abaci H. E. Biomaterials and bioengineering to guide tissue morphogenesis in epithelial organoids. Front. Bioeng. Biotechnol. 2022, 10, 1038277 10.3389/fbioe.2022.1038277. 36466337 PMC9712807

