# Supplementary material for: Different Decellularization Methods in Bovine Lung Tissue Reveals Distinct Biochemical Composition, Stiffness, and Viscoelasticity in Reconstituted Hydrogels
Source: ACS Appl Bio Mater. 2023 Feb 2;6(2):793–805. doi: 10.1021/acsabm.2c00968 (PMC9945306; doi:10.1021/acsabm.2c00968)
Supplement: Supplementary file 1 — mt2c00968_si_001.pdf [file mt2c00968_si_001.pdf]

Supporting Information

Different Decellularization Methods in Bovine Lung Tissue Reveals Distinct Biochemical Composition, Stiffness and Viscoelasticity in Reconstituted Hydrogels

Alican Kuşoğlu[1,2‡], Kardelen Yangın[1,2‡], Sena N. Özkan[1,2‡], Sevgi Sarıca[1,2‡], Deniz Örnek[1,2], Nuriye Solcan[1,2], İsmail C. Karaoğlu[6], Seda Kızılel[2,6], Pınar Bulutay[5], Pınar Fırat[5], Suat Erus[4], Serhan Tanju[4], Şükrü Dilege[4], Ece Öztürk[1,2,3*]

[‡]: These authors contributed equally.

[*]: Corresponding author; e-mail: ozturkece@ku.edu.tr

[1] Engineered Cancer and Organ Models Laboratory, Koç University, Istanbul 34450, Turkey

[2] Research Center for Translational Medicine (KUTTAM), Koç University, Istanbul 34450, Turkey

[3] Department of Medical Biology, School of Medicine, Koç University, Istanbul 34450, Turkey

[4] Department of Thoracic Surgery, School of Medicine, Koç University, Istanbul 34450, Turkey

[5] Department of Pathology, School of Medicine, Koç University, Istanbul 34450, Turkey

[6] Chemical and Biological Engineering, Koç University, Istanbul 34450, Turkey

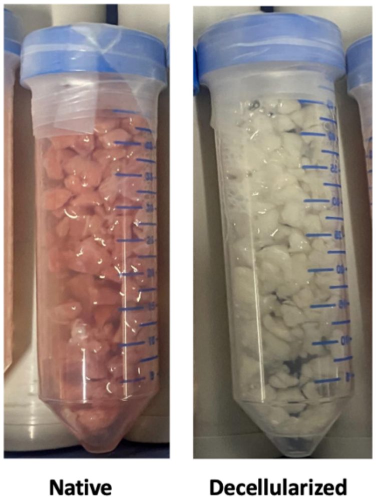

Supplementary Figure 1. Visual assessment of decellularization indicating clearance of samples, representative image for all methods.

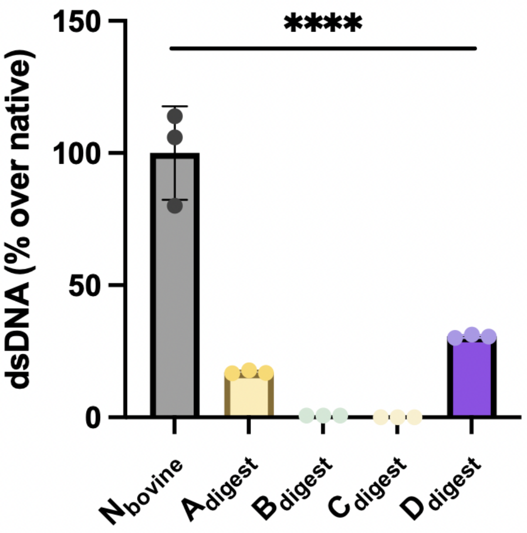

Supplementary Figure 2. dsDNA quantification after pepsin digestion of dECM powder. Error bars represent s.d. (****$p < 0.0001$).

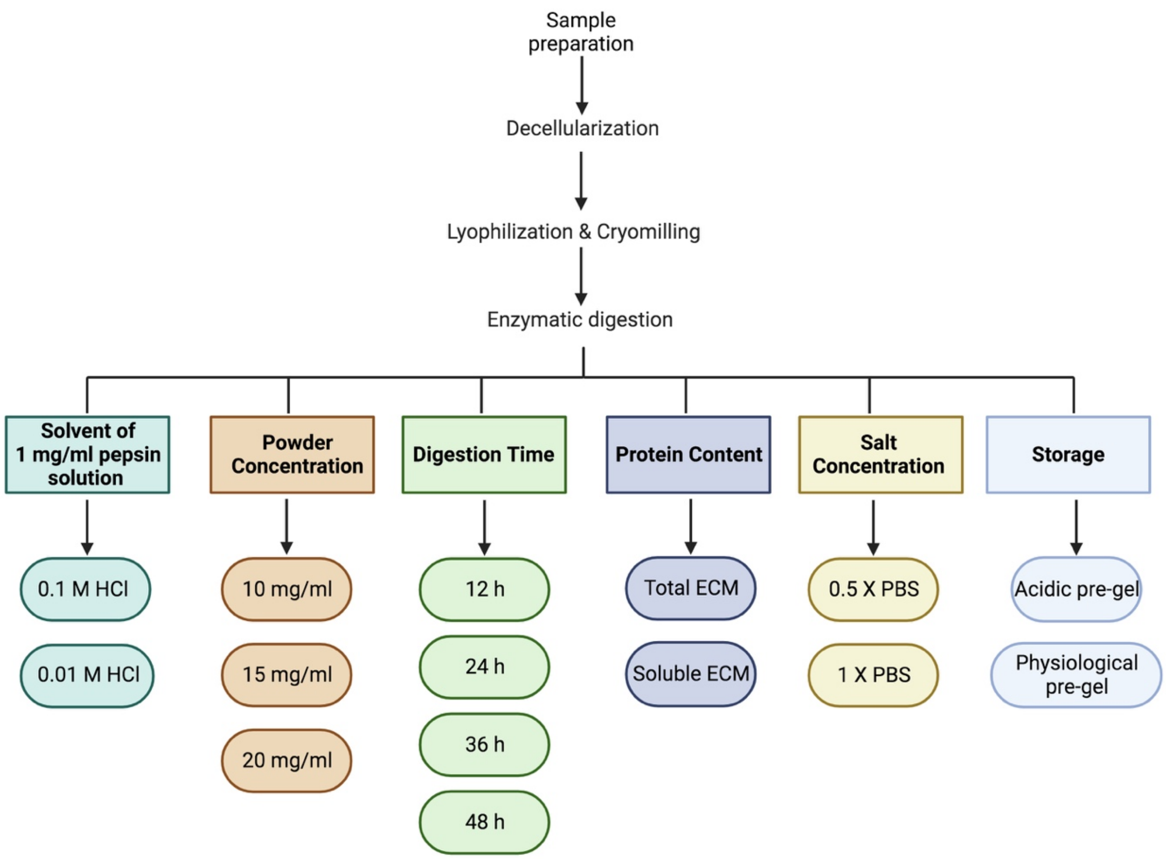

Supplementary Figure 3. Optimization parameters of the enzymatic digestion and storage conditions to achieve better gelation capacity for the hydrogels. Different concentrations of decellularized tissue powders are digested with distinct concentrations of HCl-pepsin solutions for varying time periods. Digests are stored as either with total or soluble protein components, followed by different pH and salt concentration adjustments.

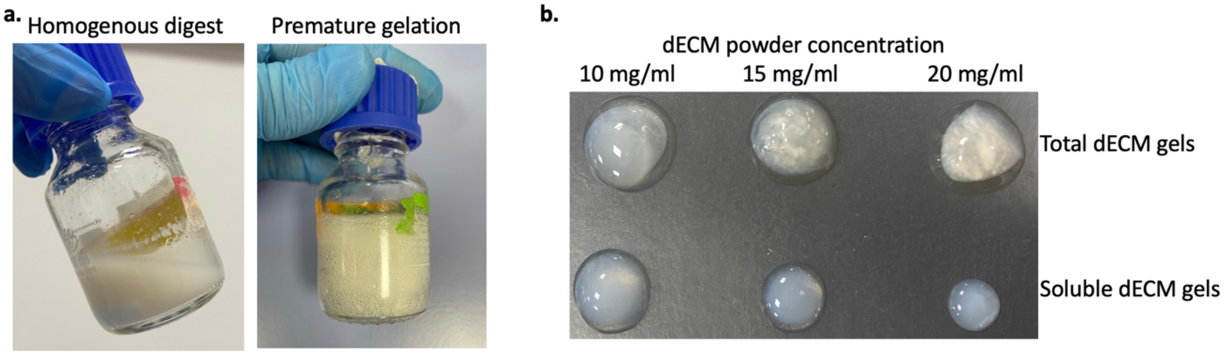

Supplementary Figure 4. a. Representative image of a normal digest (Method B) and premature gelation of 20 mg/ml dECM for all decellularization methods. **b.** Representative images of total and soluble dECM gels (Method A) with different concentrations.

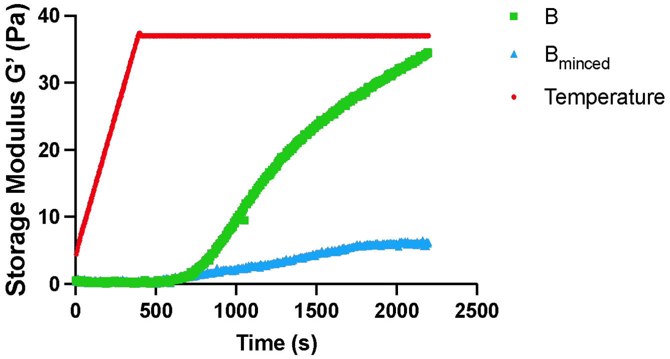

Supplementary Figure 5. Temperature ramp of dECM solutions obtained by method B with different tissue sizes prior to decellularization.

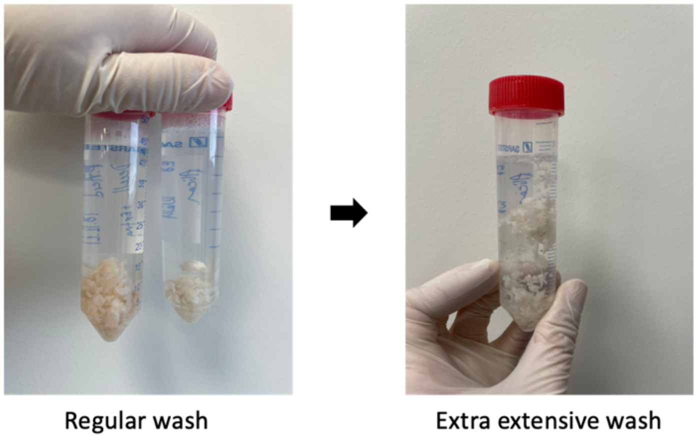

Supplementary Figure 6. Appearance of method C decellularized tissues before and after implementing an extra extensive washing process indicating the loss of remaining bubble formation and elimination of residual detergent.

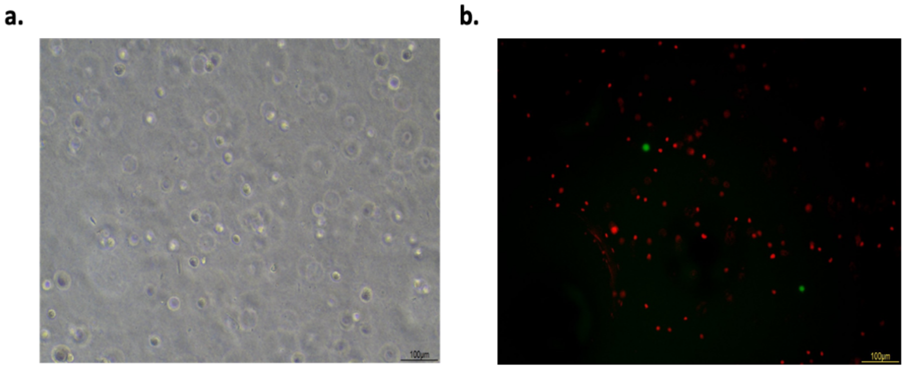

Supplementary Figure 7. a. Bright field image and **b.** Calcein-PI staining of 1 million/ml A549 cells encapsulated in C-dECM hydrogels (scale bar: 100 μm).

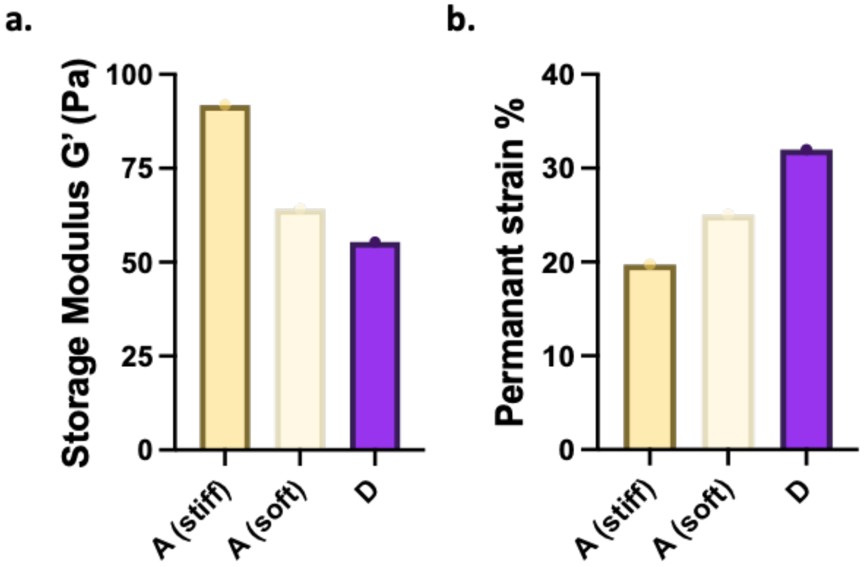

Supplementary Figure 8. a. Storage modulus of dECM hydrogels with varying ligand densities. **b.** Permanent strain preserved in dECM hydrogels with varying ligand densities.

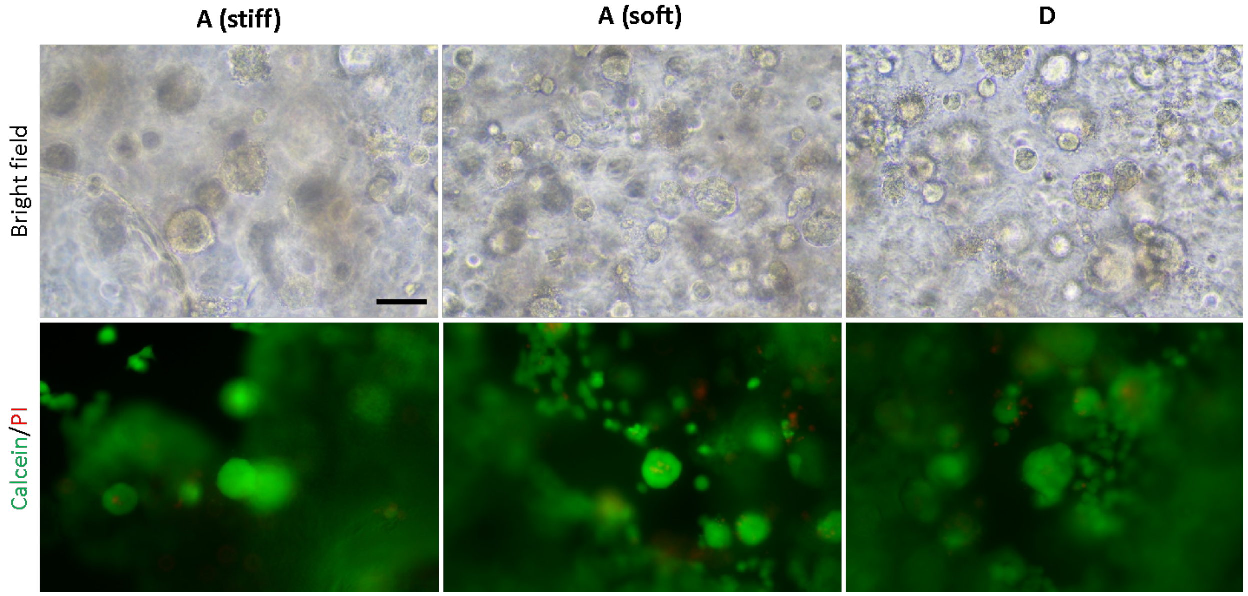

Supplementary Figure 9. Representative images of lung cancer cells encapsulated in stiff A-dECM, soft A-dECM and D-dECM. Hydrogels were stained for Calcein-AM (green) and Propidium Iodide (red) on day 10 (Scale bar: 70 μm).

Supplementary Table. Comparative summary table for all methods

**Method Name**	**A**	**B**	**C**	**D**
Decellularization Technique	Freeze-thaw cycles	Peracetic Acid	SDS	Triton-X-100
Decellularization	+	+	+	+
Residual DNA	minor	none	none	minor
Collagen content *	similar	similar	lower	similar
sGAG content*	similar	similar	similar	similar
Elastin content*	lower	similar	lower	lower
Digestion	+	+	+	+
Gelation	+	poor	+	+
Creep recovery	slower	N/A	faster	faster
Cytocompatibility	+	+	poor	+

*Collagen, sGAG and elastin contents are compared with native bovine tissue
